# Single channel approach for filtering electroencephalographic signals strongly contaminated with facial electromyography

**DOI:** 10.3389/fncom.2022.822987

**Published:** 2022-07-26

**Authors:** Carlos Magno Medeiros Queiroz, Gustavo Moreira da Silva, Steffen Walter, Luciano Brinck Peres, Luiza Maire David Luiz, Samila Carolina Costa, Kelly Christina de Faria, Adriano Alves Pereira, Marcus Fraga Vieira, Ariana Moura Cabral, Adriano de Oliveira Andrade

**Affiliations:** ^1^Centre for Innovation and Technology Assessment in Health, Federal University of Uberlândia, Uberlândia, Brazil; ^2^Department of Medical Psychology, Clinic of Psychosomatic Medicine and Psychotherapy, University Hospital Ulm, Ulm, Germany; ^3^Bioengineering and Biomechanics Laboratory, Federal University of Goiás, Goiânia, Brazil

**Keywords:** EEG, EMG, adaptive filtering, signal decomposition, facial electromyography

## Abstract

Eliminating facial electromyographic (EMG) signal from the electroencephalogram (EEG) is crucial for the accuracy of applications such as brain computer interfaces (BCIs) and brain functionality measurement. Facial electromyography typically corrupts the electroencephalogram. Although it is possible to find in the literature a number of multi-channel approaches for filtering corrupted EEG, studies employing single-channel approaches are scarce. In this context, this study proposed a single-channel method for attenuating facial EMG noise from contaminated EEG. The architecture of the method allows for the evaluation and incorporation of multiple decomposition and adaptive filtering techniques. The decomposition method was responsible for generating EEG or EMG reference signals for the adaptive filtering stage. In this study, the decomposition techniques CiSSA, EMD, EEMD, EMD-PCA, SSA, and Wavelet were evaluated. The adaptive filtering methods RLS, Wiener, LMS, and NLMS were investigated. A time and frequency domain set of features were estimated from experimental signals to evaluate the performance of the single channel method. This set of characteristics permitted the characterization of the contamination of distinct facial muscles, namely Masseter, Frontalis, Zygomatic, Orbicularis Oris, and Orbicularis Oculi. Data were collected from ten healthy subjects executing an experimental protocol that introduced the necessary variability to evaluate the filtering performance. The largest level of contamination was produced by the Masseter muscle, as determined by statistical analysis of the set of features and visualization of topological maps. Regarding the decomposition method, the SSA method allowed for the generation of more suitable reference signals, whereas the RLS and NLMS methods were more suitable when the reference signal was derived from the EEG. In addition, the LMS and RLS methods were more appropriate when the reference signal was the EMG. This study has a number of practical implications, including the use of filtering techniques to reduce EEG contamination caused by the activation of facial muscles required by distinct types of studies. All the developed code, including examples, is available to facilitate a more accurate reproduction and improvement of the results of this study.

## 1. Introduction

Electroencephalography is a technique used to record the activity on the scalp of measured cerebral cortex neuronal populations. It is derived from a high temporal resolution, non-invasive macroscopic process and is a low-cost method compared to a functional neuroimaging test (Pivik et al., 1993; McMenamin et al., 2011; Mamun et al., 2013). The electroencephalogram (EEG) is widely used in a variety of clinical and commercial applications, including cognitive neuroscience, brain-skill quantification, pathological diagnosis, biometrics, and Brain-Computer Interfaces (BCIs) (Foxe and Snyder, 2011; Abo-Zahhad et al., 2015; Mihajlovic et al., 2015; Ramadan and Vasilakos, 2017).

The system for measuring EEG amplifies the tiny disturbances of the electrical potentials of the electrodes positioned on the scalp, which is anatomically separated from the signal-generating sources by the meninges, skull bones, and scalp. Thus, the synaptic potentials which usually have low amplitudes, in the order of millivolts, are strongly attenuated by these anatomical structures, reducing the amplitude of the signals recorded at the scalp (Hero, 2006). Due to this low amplitude, which typically does not exceed 100 μ*V*, the EEG signal is highly susceptible to artifacts. These artifacts are usually caused by electromagnetic fields generated by nearby electronic devices and the power grid. In addition, artifacts can be produced by other sources of electrophysiological signals, e.g., muscular and heart activity or eye movement (Sweeney et al., 2012; Urigüen and Garcia-Zapirain, 2015). This contamination decreases the performance of applications such as BCI and diagnosis of pathological disfunctions, since the amplitude of the artifact will typically be several orders of magnitude greater than the EEG amplitude (Nunez and Srinivasan, 2006; Tatum et al., 2011).

In this context, the characterization and elimination of artifacts is relevant for the correct interpretation and use of EEG. Facial electromyographic (EMG) signals are a primary source of EEG contamination. The main challenge with respect to the elimination of noise generated by the EMG signal lies in the fact that EMG emerges from the anatomically positioned muscles along the skull. Even weak muscular contractions can be detected throughout the scalp due to the phenomenon of conductive volume. In addition, the EMG signal overlaps the spectrum of the EEG signal in virtually all frequency bands (Goncharova et al., 2003).

To solve this problem, several EEG filtering methods are described in the literature. However, these methods have some limitations, mainly related to the inability to completely remove noise from the corrupted signal without the introduction of undesired distortions, and the need for *a priori* noise information for signal filtering. These limitations, associated with several features estimated from the EEG signal to suit the diversity of applications, motivate the search for multiple gold standards for removing EEG artifacts (Safieddine et al., 2012; Gabsteiger et al., 2014; Urigüen and Garcia-Zapirain, 2015; Bono et al., 2016; Upadhyay et al., 2016; Frølich and Dowding, 2018; Mucarquer et al., 2020).

Frequency selector filters, such as a linear Butterworth filter, are one of the main techniques described in the literature for the removal of physiological artifacts from EEG. However, the use of such filter class is only effective when the frequency range of the signal and noise are not overlapped (Sweeney et al., 2012).

The literature suggests the use of single-channel techniques for muscular artifact removal from EEG instead of multichannel techniques, e.g., Independent Component Analysis (ICA) and Canonical Correlation Analysis (CCA). The following methods are commonly employed for this purpose: adaptive filtering (Correa et al., 2007; Diniz, 2008; Kher and Gandhi, 2016); Wiener filtering (Maki et al., 2015; Ferdous and Ali, 2017), Bayesian filtering (Morbidi et al., 2008), Blind Source Separation (BSS) (James and Hesse, 2004; Albera et al., 2012), wavelet transform (WT) (Ngoc et al., 2015; Turnip and Pardede, 2017), Empirical Mode Decomposition (EMD) (Gaur et al., 2015; Alam and Samanta, 2017), and the combination of these techniques, i.e., hybrid methods (Chen et al., 2014; Daly et al., 2015; Salsabili et al., 2015; Bono et al., 2016; Zeng et al., 2016).

An adaptive filter is required when fixed specifications are unknown. The literature describes that the most prevalent family of algorithms for removing EEG artifacts is based on the method of least squares (Correa et al., 2007; Kim and Kim, 2018). Adaptive filters vary in time because their parameters are continuously changing to meet a performance requirement (Gerardo et al., 2011).

Wiener filtering is appropriate in situations in which the signal and noise spectrum are overlapping, although it requires an estimated, measured, or reliable reference to operate. Sengupta and Kay (1995) showed that the performance of the multichannel Wiener filter (MWF) outperformed that of BSS for removal of EEG artifacts of various types, i.e., those that were annotated as unwanted by the user. In addition, Ferdous and Ali (2017) compared Wiener and Kalman filters, and again the Wiener filter was more effective for removing EEG artifacts. However, the Wiener filter was mainly applied to remove ocular artifacts, not including muscular artifacts with low SNR, i.e., lower than −10 dB.

Gao et al. (2010) employed an adaptive algorithm to remove ECG from EEG during sleep apnea records by means of Discrete Wavelet Transform (DWT). Iyer and Zouridakis (2007) compared DWT with an ICA filter for subsequent detection of single-trial evoked potential. Krishnaveni et al. (2006) compared the Joint Approximation Diagonalization of Eigen-matrices (JADE) algorithm (Rutledge and Bouveresse, 2013) with DWT for the removal of EOG from EEG.

Empirical Mode Decomposition (EMD) was successfully used for the removal of EEG artifacts in Safieddine et al. (2012) and Zhang et al. (2012) and also in conjunction with BSS methods (Zhang et al., 2012; Sweeney, 2013). A broad review of the application of EMD and its variations on EEG signal processing is given in Sweeney-Reed et al. (2018).

Recent efforts have been focused on the combination of these algorithms for removing artifacts from the EEG. Hybrid methods are, therefore, considered the state of the art in EEG filtering because they use the advantages of different methods in two or more stages and have presented the best results in their applications (Castellanos and Makarov, 2006; Sweeney et al., 2012; Sweeney, 2013; Urigüen and Garcia-Zapirain, 2015; Bono et al., 2016; Mannan et al., 2016; Zeng et al., 2016; Frølich and Dowding, 2018). The main combinations of algorithms in different filtering stages are: (i) adaptive filtering with BSS-ICA; (ii) EMD with BSS; (iii) wavelet with BSS; (iv) adaptive filtering with EMD (Mannan et al., 2016).

Currently, single-channel techniques have been shown to be the most effective approach for the removal of facial muscular artifacts from EEG, especially when a reference signal is known (Chen et al., 2016). However, the main limitation of this class of noise removal technique is that its performance is low for signal-to-noise ratios below −10 dB (Chen et al., 2016; Zeng et al., 2016; Mucarquer et al., 2020), which is typical in EEG contaminated by facial electromyography. To the best of our knowledge, there is lack of studies addressing the removal of facial muscular artifacts from EEG. This is important when there is a need to monitor brain activity during human computer interaction (Andrade et al., 2013).

To contribute to the research on facial EMG removal from EEG, this study presents an EEG filtering approach for facial EMG removal that is independent of an external reference noise and suited for low SNR signals. The strategy involves determining a single channel reference signal using a decomposition technique, which is subsequently utilized by an adaptive filter to attenuate facial EMG. The reported results consider the evaluation of various decomposition and adaptive filtering methods, as well as the evaluation of filtering performance in the time and frequency domains. The experimental protocol used in this study is based on the practical need to assess brain activity for motor learning quantification during interaction with a myoelectric interface (Andrade et al., 2013).

## 2. Materials and methods

### 2.1. Experimental protocol

Data were collected from ten healthy individuals during the execution of successive facial muscular contractions to characterize the EEG signal contamination by facial muscular activity. This experimental protocol was based on previous published work (Andrade et al., 2013) reporting the implementation of a facial EMG interface and motor learning assessment.

This study followed the Resolution 466/2012 of the National Health Council. The study was conducted at the Centre for Innovation and Technology Assessment in Health of the Federal University of Uberlândia (UFU), Brazil. The experimental protocol was approved by the Human Research Ethics Committee (CEP-UFU), CAAE Number: 43670815.4.0000.5152.

**Figure 1:**
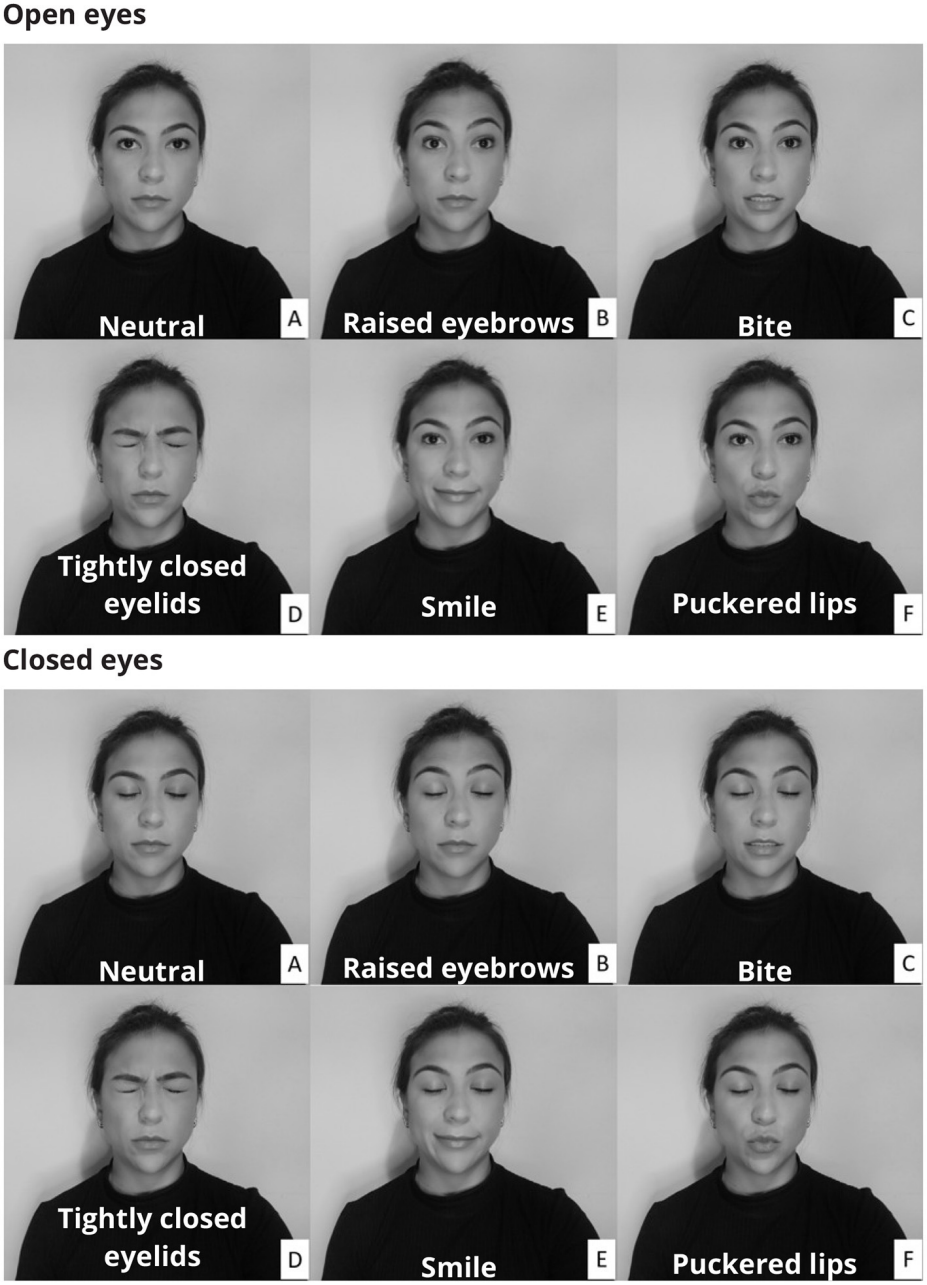
Participants were instructed to make a variety of facial expressions by activating muscles whose electrical activity corrupts the electroencephalogram. The facial expressions were performed with both open and closed eyes. In the neutral condition **(A)** there was no muscular contraction, whereas in the other conditions the following muscles were activated: Frontalis **(B)**, Masseter **(C)**, Orbicularis Oculi **(D)**, Zygomatic **(E)**, and Orbicularis Oris **(F)**.

The protocol consisted of two sets of facial muscle contractions, one with the eyes open and one with the eyes closed. The open and closed eyes conditions allowed for the evaluation of the filtering methods considering changes in the EEG amplitude. The participants were instructed to perform a series of facial expressions (Figure 1) by contracting five distinct muscles (Frontalis, Masseter, Orbicularis Oculi, Orbicularis Oris, and Zygomatic).

**Figure 2:**
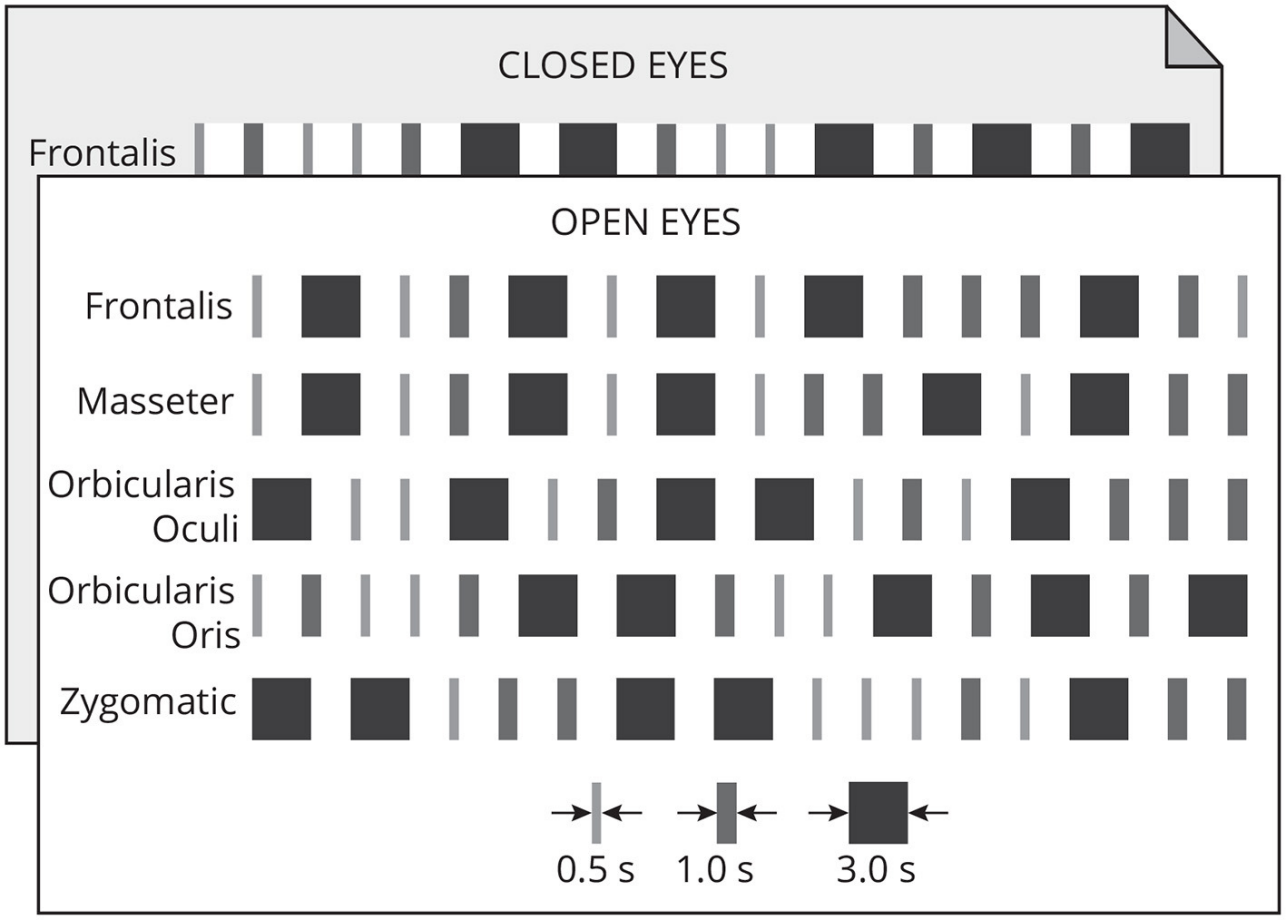
Each muscle was contracted 15 times in one of three timing patterns: long (3 s), medium (1 s), and short (0.5 s). Each pattern of contraction was repeated five times randomly. A 2-s neutral period followed each contraction. This protocol was executed with open and closed eyes.

Each muscle was contracted 15 times following a random timing protocol (Figure 2) of three distinct patterns: long (3 s), medium (1 s), and short (0.5 s). The onset and duration of the contractions were controlled by an auditory stimulus (beep). The volunteer was asked to maintain the contraction while listening to the beep, and to finish the contraction immediately after the auditory stimulus considering these timing patterns. There were five repetitions of each contraction pattern. Each contraction was followed by a 2 s neutral period. Each participant performed 150 muscle contractions.

Thus, for 10 participants, the data set consisted of 500 contractions lasting 3 s, 500 contractions lasting 1 s, and 500 contractions lasting 0.5 s, for a total of 2,250 s of EEG signals corrupted by facial EMG.

In this study the bipolar, i.e., differential, EEG montage was used to deliberately differentiating potentials between spatially adjacent locations as this may lead to improved signal-to-noise ratio of the collected signal. This type of configuration is also known as longitudinal configuration and widely employed in clinical practice (Kutluay and Kalamangalam, 2019). Although the employed montage was bipolar, the electrodes were positioned by using an EEG cap following the 10-20 International system of EEG electrode placement.

EMG signals were detected by using disposable sensors (Meditrace, USA) and collected simultaneously to EEG by using the RHD USB interface board (lntan, USA). The signals were sampled at 5 kHz and band-pass filtered (0.1 Hz–1 kHz).

### 2.2. Signal processing stages for the implementation of a single channel approach to EEG filtering

**Figure 3:**
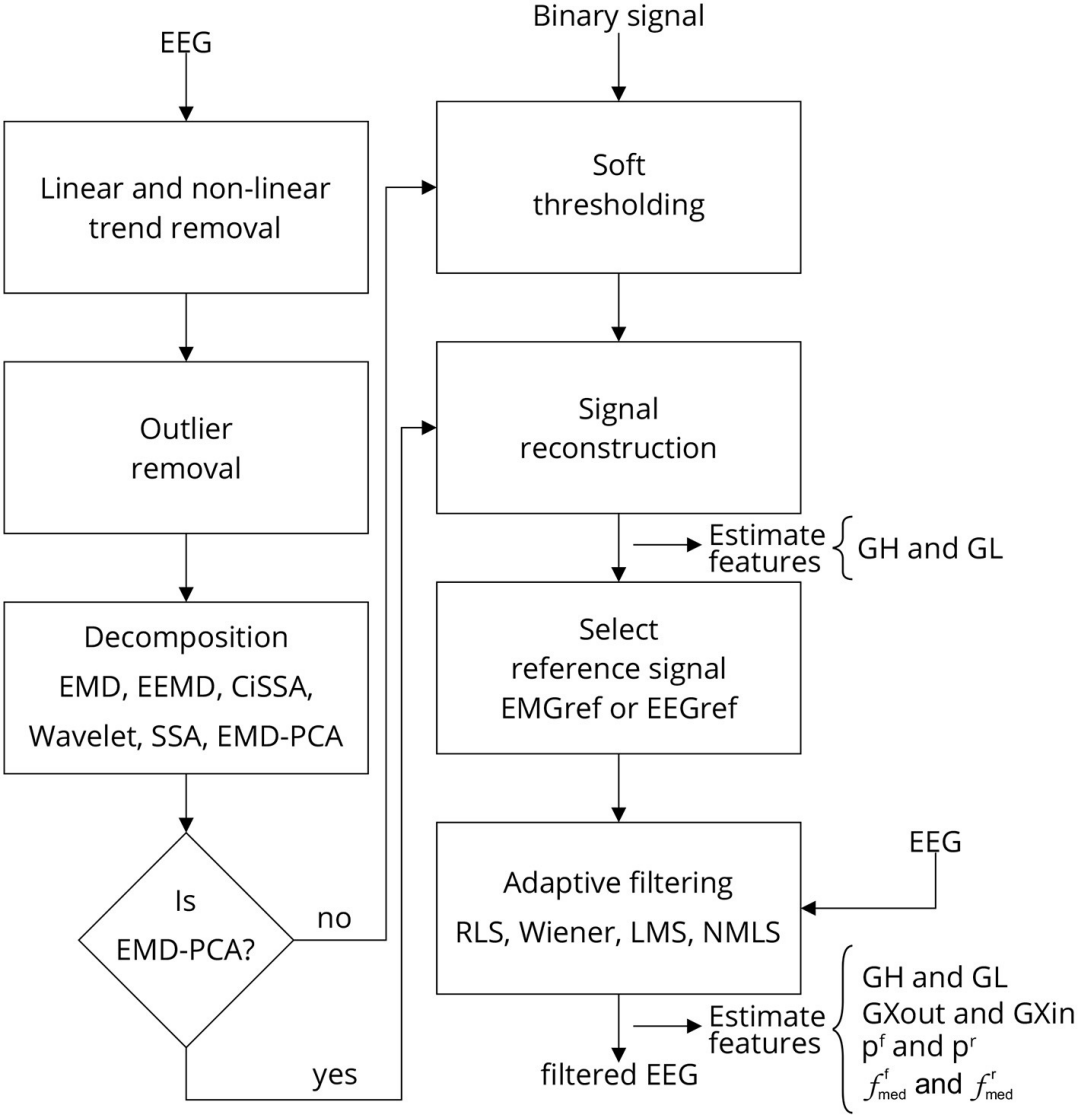
Filtering EEG signals corrupted by EMG *via* a series of steps. Linear and non-linear trends are eliminated, as well as outliers. The signal is then decomposed using one of the methods outlined, and the resulting components are thresholded. The thresholding process requires the identification of noise periods in the signal, which are provided by a binary signal generated by an EMG burst detector (Figure 4). Once the components have been thresholded, the filtered signal is reconstructed, producing a reference signal, i.e., EEG or EMG reference signal, which can be used as a reference for one of the indicated adaptive filters. Various characteristics are estimated to evaluate the filtering process at distinct stages. Note that when the method EMD-PCA is used it is not necessary to execute the soft-thresholding stage.

Figure 3 depicts the sequence of steps required to implement a single approach for filtering EEG signals corrupted by facial EMG. The first step is to eliminate any linear and non-linear trends from the collected signals. These tendencies are typically due in part to drift caused by changes in the impedance between the skin and the electrode, as well as cable and skin motion. By fitting a linear model to the time series and then subtracting the resulting straight line from the data, the linear trend is eliminated.

The non-linear trend is estimated by applying a sliding, non-overlapping, rectangular window of 20 ms (100 samples) to the data and then estimating the median of each window. The resultant time-series is interpolated using a Piecewise Cubic Hermite Interpolating Polynomial (pchip) so that it can be re-sampled with the same number of samples as the input time-series. The resultant signal is the non-linear trend that should be subtracted from the signal (i.e., the electroencephalogram or electromyogram). Supplementary Figure 1, which is available as Supplementary Material, shows an example of the result of this signal processing stage applied to an acquired EMG signal.

Outliers can result from any sudden abnormal changes in data amplitude that exceed or fall below predetermined thresholds. In this study, the upper/lower threshold was established as the mean plus/minus ten times the standard deviation of the data in the EMG-contaminated regions. The outliers were replaced by random scalars drawn from the standard normal distribution.

The pre-processed signal is then decomposed by one of the following decomposition methods: Empirical Mode Decomposition (EMD) (Huang et al., 1998), Extended Empirical Mode Decomposition (EEMD) (Wu and Huang, 2009), Circulant Singular Spectrum Analysis (CiSSA) (Bógalo et al., 2021), Wavelet Decomposition (Turnip and Pardede, 2017), or Singular Spectrum Analysis (SSA) (Bógalo et al., 2021). For the methods EMD, EEMD, CiSSA, and Wavelet, the maximum number of components was set to 10. For EMD and EEMD, the pchip interpolation method was utilized. For the EEMD approach, there were five ensembles. For Wavelet Decomposition, the mother wavelet was *coif5*. For the method SSA, the window length was 100 and the proportion of explained variance was 80%.

The obtained components are then soft-thresholded to eliminate noise as explained in a previous work (Andrade et al., 2006). For each signal component *C* = {*c*_1_, *c*_2_, ⋯ , *c*_*M*_}, a threshold, *t*_*m*_|*m* = {1, ⋯ , *M*}, is estimated, and soft-thresholding is applied to individual components as shown in Equation (1),


(1) $$\begin{array}{l} tc_{m}=sign\left(c_{m}\right){\left(|c_{m}|-t_{m}\right)}_{+} \end{array}$$

where *tc*_*m*_ is the de-noised (or thresholded) version of the *mth* signal component and the function (*x*)_+_ is defined as


(2) $$\begin{array}{l} {\left(x\right)}_{+}=\{\begin{array}{cc} 0, & x<0\\ x, & x>=0. \end{array} \end{array}$$

**Figure 4:**
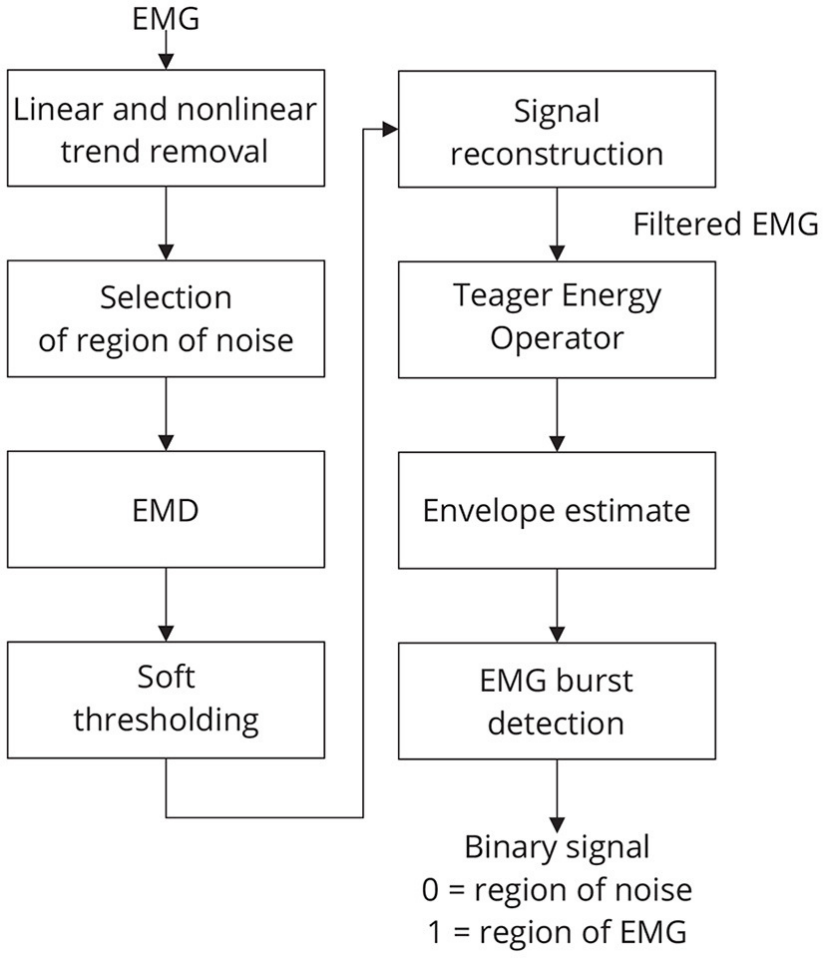
Sequence of required steps for detecting EMG bursts. First, the input signal is preprocessed by removing linear and nonlinear trends, and then the resulting signal is decomposed using EMD. The estimated components are soft-thresholded using *a priori* knowledge of the signal's noise level. Signal filtration is achieved by summing the thresholded components. The EMG envelope is determined by estimating the signal's energy, and bursts are detected using a threshold. As a result of this step, a binary signal is generated in which low levels indicate noise and high levels indicate EMG activity.

The threshold *t*_*m*_ is estimated by using the following strategy: a window of noise is selected from the original signal and then the boundaries of this window are used to extract regions of noise from the signal components. For noise information selection, a binary signal is used. Low-level periods in this binary signal correspond to noise, while high-level periods correspond to EMG regions. Figure 4 provides an overview of the required steps for automatic EMG burst detection (Andrade et al., 2006). Although the EMG signal is the input signal illustrated in Figure 4, EMG bursts can be detected directly from EEG that has been corrupted by EMG. Because EMG signals were collected simultaneously with EEG, we decided not to use the EMG-corrupted EEG in this study for EMG burst detection.

The standard deviation of each of those regions is then estimated, multiplied by a constant *k*, to obtain the required thresholds (*t*_1_, ..., *t*_*M*_). A typical value of *k* is 1.5 (Andrade et al., 2006). It is possible to vary *k* to control the signal filtering.

The EMG reference signal is obtained during the signal reconstruction stage. The adaptive filter may use this signal as a reference. Optionally, an EEG signal may be used as a reference. In this case, the noise-corrupted EEG is subtracted from the EMG reference signal to produce the EEG reference signal. Note that the reference signal is a filtered signal, resulting from the reconstruction of soft-thresholded signal components. In the case of the EMD-PCA method, the signal is reconstructed by selecting the principal components that account for at least 80% of the data variability. For the other methods, the signal is reconstructed based solely in the estimated components.

The reference signal is sent through an adaptive filter, which removes EMG noise from the electroencephalogram. One of the following adaptive filters can be chosen: Recursive Least Squares (RLS), Wiener filter, Least Mean Square (LMS), and Normalized Least Mean Square (NMLS) (Farhang-Boroujeny, 1999). Except for the method NMLS, which had an order of 4, the step-size utilized for all filters was 10^−7^ and the order was 10. Matlab R2022a was used to implement all of the code required for signal processing (MathWorks, USA).

### 2.3. Estimate of features for filtering assessment

Several features were estimated to enable for the characterization of EMG contamination on EEG and to compare different approaches used in the investigation. The stages in which the set of features is estimated are depicted in Figure 3.

#### 2.3.1. Time domain features

**Figure 5:**
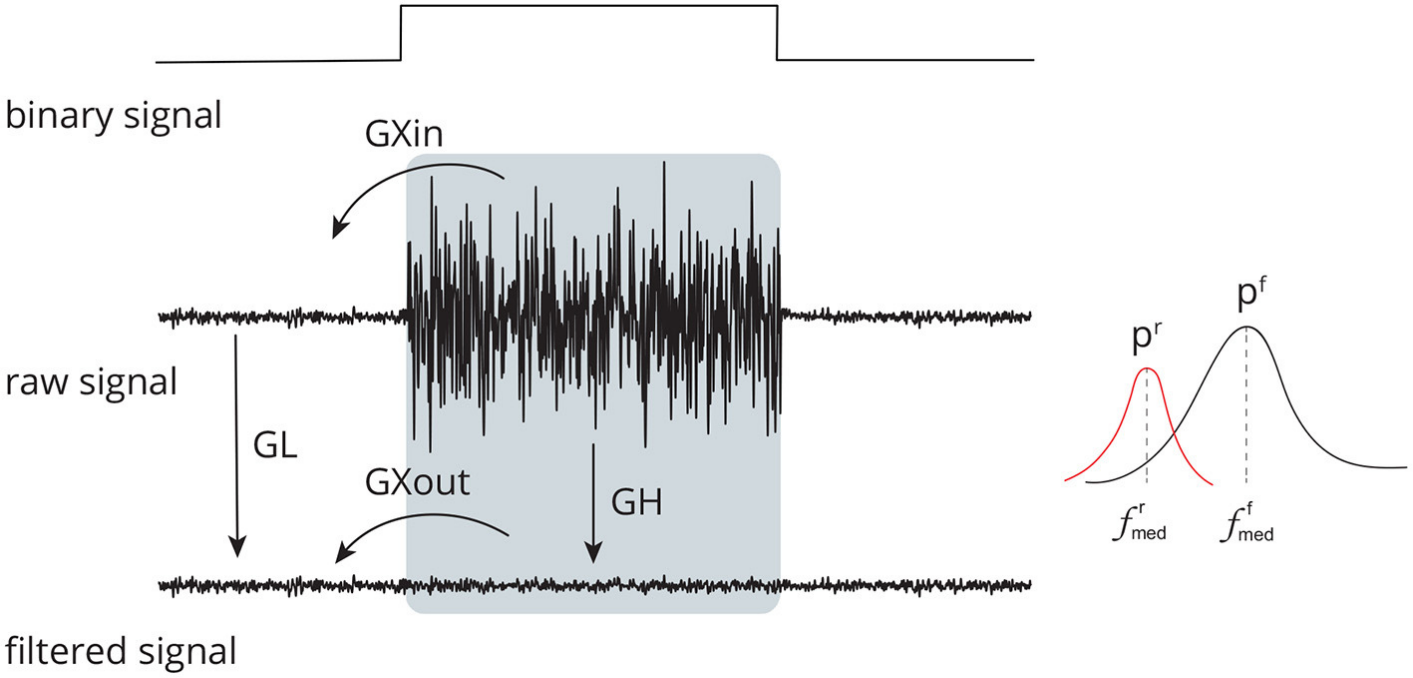
Set of time (*GL*, *GH*, *GXin*, and *GXout*) and frequency (*p*^*r*^, *p*^*f*^, $f_{med}^{r}$, and $f_{med}^{f}$) domain features used to evaluate the performance of distinct methods for adaptive filtering EEG corrupted by facial EMG. The features compare the signals before and after adaptive filtering and consider the regions in which there is the presence and absence of muscle activity.

The feature *GL* assesses the effect of filtering in regions with no EMG activity, i.e., regions with a low binary signal (Figure 5). Any filtering method is expected to preserve the amplitude and shape of the signals in this region as much as possible.

Equation (3) defines *GL*. The general idea is to apply a sliding, non-overlapping 1 s window with 5,000 samples to the data, compute the root-mean-square (RMS) value for each window, and then estimate the median of the RMS values. As the length of the window is 1 s, the number of samples in each window is equal to the sampling frequency *f*_*s*_, which is 5 kHz.


(3) $$\begin{array}{l} GL=20\log\left(Xout_{0}^{\prime }/Xin_{0}^{\prime }\right) \end{array}$$

where


(4) $$\begin{array}{l} Xin_{0}^{\prime }=median\left\{RMS\left(Xin_{0}{|}_{{i_{1}}_{n}}^{{i_{2}}_{n}}\right)\right\} \end{array}$$

corresponds to the median of the RMS values estimated from the signal *Xin*_0_, i.e., the non-filtered signal, and


(5) $$\begin{array}{l} Xout_{0}^{\prime }=median\left\{RMS\left(Xout_{0}{|}_{{i_{1}}_{n}}^{{i_{2}}_{n}}\right)\right\} \end{array}$$

corresponds to the median of the RMS values estimated from the signal *Xout*_0_, i.e., the filtered signal, being $n=\left\{1,2,\cdots \;,\lfloor \frac{N}{f_{s}}\rfloor \right\}\in \mathbb{N}$ a set in which each of its values corresponds to a window, $\left\lfloor \frac{N}{f_{s}}\right\rfloor$ the total number of windows, $i_{1}=\left\{1,f_{s}+1,2f_{s}+1,\cdots \;,\left(\lfloor \frac{N}{f_{s}}\rfloor f_{s}+1\right)-f_{s}\right\}\in \mathbb{N}$ the discrete time in which the window starts, and $i_{2}=\left\{f_{s}+1,2f_{s}+1,\cdots \;,\left\lfloor \frac{N}{f_{s}}\right\rfloor f_{s}+1\right\}\in \mathbb{N}$ the discrete time in which the window ends. *N* is the number of samples of the signal.

*GH* (Figure 5) is the feature that estimates the ratio between noise-corrupted and filtered signals in an EMG-contaminated region. It is defined in Equation (6). It is calculated in a manner similar to that of *GL*; hence, equivalent definitions will not be supplied to prevent duplication.


(6) $$\begin{array}{l} GH=20\log\left(Xout_{1}^{\prime }/Xin_{1}^{\prime }\right) \end{array}$$

While the *GL* and *GH* features evaluate the ratio of signal amplitudes considering different parts of the binary signal, the *GXin* and *GXout* features measure the ratio of signal amplitudes comparing regions with and without noise (Figure 5), as given in Equations (7) and (8). The estimates are similar to that of *GL* and *GH*, thus they are not provided.


(7) $$\begin{array}{l} GXin=20\log\left(Xin_{1}^{\prime }/Xin_{0}^{\prime }\right) \end{array}$$

(8) $$\begin{array}{l} GXout=20\log\left(Xout_{1}^{\prime }/Xout_{0}^{\prime }\right) \end{array}$$

#### 2.3.2. Frequency domain features

For the estimate of the frequency domain features (*p*^*r*^, *p*^*f*^, $f_{med}^{r}$, and $f_{med}^{f}$), first the power spectral density estimate, *pxx*, of the discrete-time signal was estimated by using the Yule-Walker method. The signal energy was estimated for the frequency *f* = {0, 0.01, 0.02, ⋯ , *f*_*s*_/2} in Hz, considering a model of order 10. The median frequency and its corresponding energy were estimated from *pxx* for the non-filtered ($f_{med}^{r}$ and *p*^*r*^) and filtered ($f_{med}^{f}$ and *p*^*f*^) signals.

### 2.4. Statistical analysis

Statistical analysis was performed using R, which is a language and environment for statistical computing (R Core Team, 2021). Considering the studied methods and experimental conditions, the analyses were designed to answer the following research questions: (i) Which facial muscle contributes the most to EEG contamination? (ii) Which decomposition methods are preferable for generating reference signals for adaptive filtering? (iii) Considering its effect on the EEG signal and its components, which adaptive filtering methods are the most desirable?

#### 2.4.1. Characterization of the contamination of the electroencephalogram by distinct facial muscles

**Figure 6:**
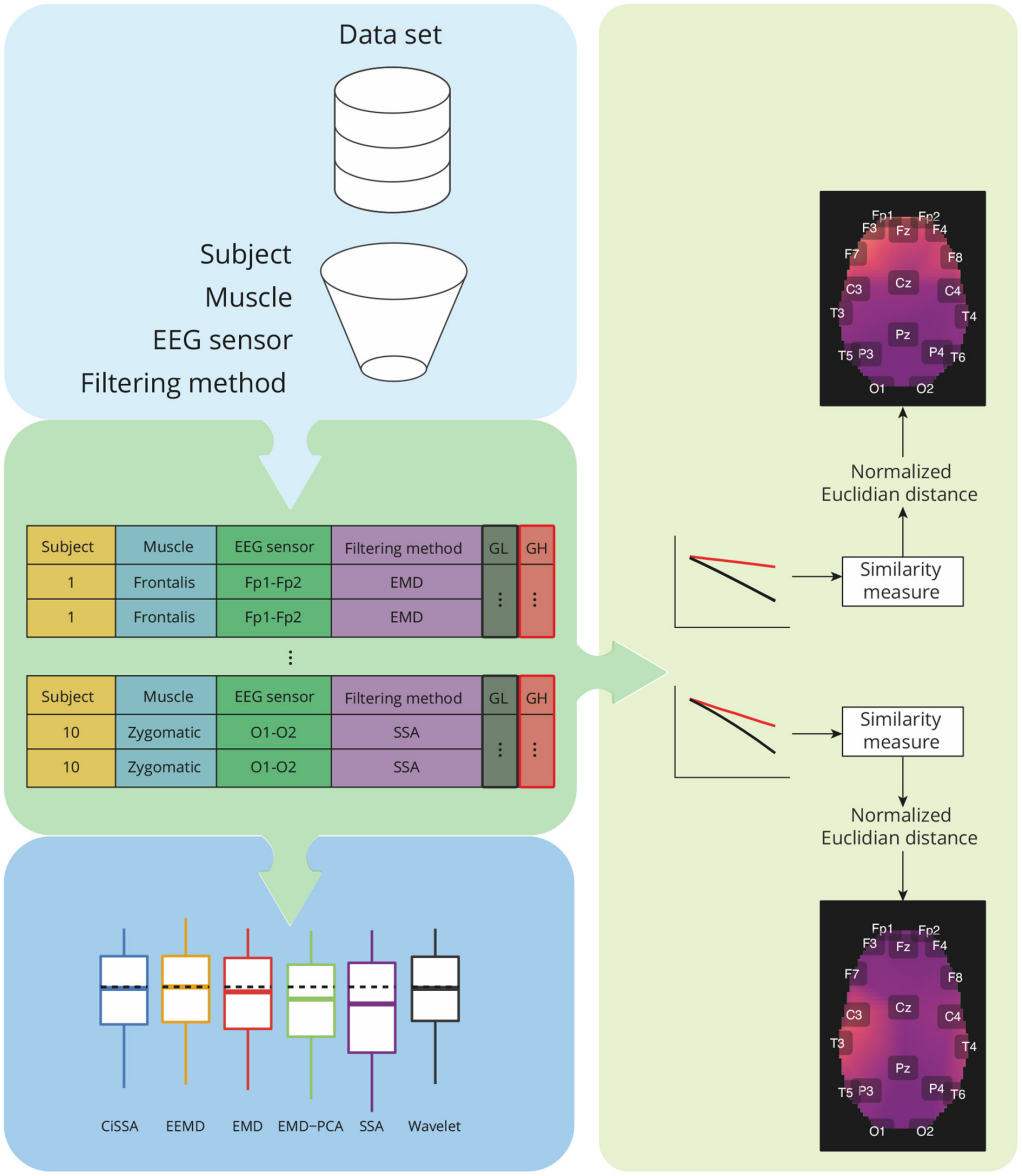
Overview of the analysis required for characterization of contamination caused by different facial muscles, as well as a comparison of the performance of decomposition methods based on the features *GH* and *GL*. The analysis takes into account data grouping by participants, muscles, EEG sensors, and filtering methods. For each group, a similarity measure based on the normalized Euclidian distance can be estimated between a pair of vectors representing *GH* and *GL* estimates for varying a parameter used in the soft-thresholding of the signal components. The similarity metrics are used to create spatial brain maps that depict the contamination of EMG levels at various areas. Statistical analyses are carried out for *GH*, *GL*, and similarity measures.

**Figure 7:**
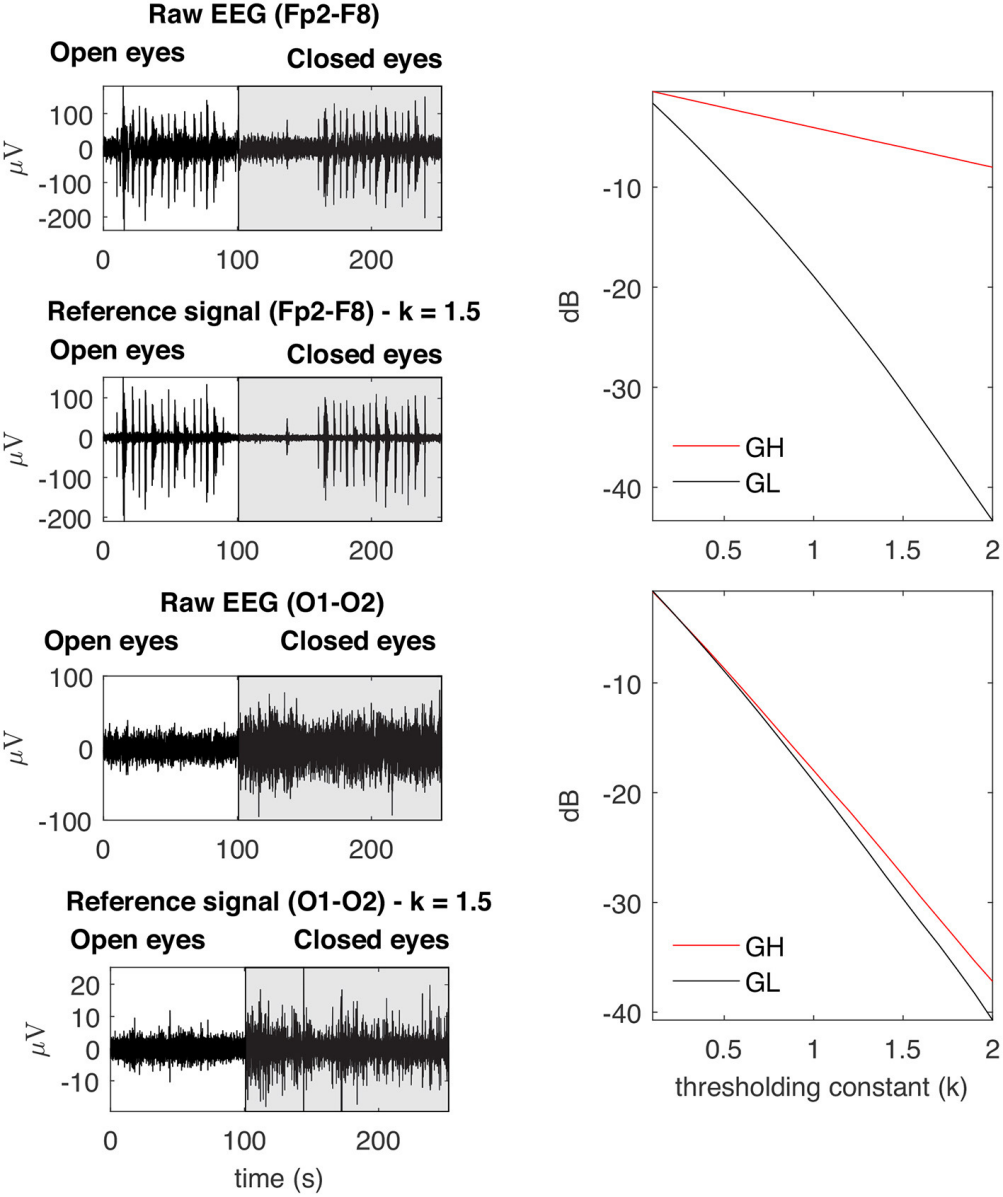
Examples of typical *GL* and *GH* feature vectors obtained for high (Fp2–F8) and low (O1-O2) levels of EMG contamination on EEG signals. By decomposing the raw EEG signals with EMD, the reference signals were obtained. The contamination level of the EEG signal can be captured by the distance between the feature vectors, in the sense that an increased distance is related to a lower signal to noise ratio. The examples demonstrate typical collected signals for the open and closed eyes scenarios.

Figure 6 depicts the main steps employed to characterize the contamination of EEG by facial muscles. The *GL* and *GH* features were used to investigate how distinct facial muscles contaminate EEG signals. By varying the parameter *k* of the soft-thresholding procedure, from 0.1 to 2.0 with a resolution of 0.1, it is possible to obtain, for each decomposition method, a distinct feature vector for *GL* and *GH*. Figure 7 shows typical vectors for the collected signals.

The estimated features, *GL* and *GH*, were grouped by subjects, muscles, EEG sensors, and filtering methods. For each pair of feature vectors a similarity measure based on the normalized Euclidian distance was computed (Barrett, 2005). These values of the similarity measures were used to generate a topological map in which the light colors are associated to the contamination of facial EMG on EEG. A customized function was developed in R to generate the topological maps according to the Montreal Neurological Institute (MNI) coordinates mapped to the International 10-20 System (Okamoto et al., 2004).

In addition to the visualization of topological maps, the normalized Euclidean distance between *GL* and *GH* were used to quantify the electromyographic contamination produced by distinct muscles. The box plot of the normalized Euclidean distance were computed and the mean of the variables were statistically compared. The statistical analysis was performed considering the scenarios of independence and dependence to the subjects.

To compare the variables, one-way analysis of variance (ANOVA) was used. After fitting the ANOVA model to the data, the model's assumptions were verified, i.e., the evaluation of the homogeneity of variances (Levene's test) and normality of the residuals' distribution (Kolmogorov–Smirnov test). The *p*-value for all analyses was 0.05. Tukey's honestly significant difference test (Tukey's HSD) was used to examine the significance of differences between sample means. If the variables did not meet the assumptions of ANOVA, the Kruskal–Wallis rank sum test and Dunn's test for multiple comparisons were employed to compare them.

#### 2.4.2. Comparison of the performance of distinct decomposition methods

As seen in Figure 3, the decomposition methods in conjunction with the soft-thresholding procedure act as a filtering method that enables the generation of a suitable reference signal for adaptive filtering. In this regard, it is necessary to preserve EMG regions as much as possible so that adaptive filters can attenuate them appropriately.

The *GL* and *GH* measures were used to compare the performance of different decomposition approaches. Based on the definitions of *GL* (Equation 3) and *GH* (Equation 6), the most appropriate filtering method is the one that produces the lowest *GL* and the largest *GH*, i.e., the method that reduces the signal amplitude in the regions without EMG while preserving the amplitude in the regions of EEG contaminated by EMG as much as possible. For the generation of an appropriate reference signal, a substantially greater reduction of *GL* relative to *GH* is expected.

The box plots of *GL* and *GH* were generated, and their respective means were compared statistically. For this purpose, the statistical analysis followed the procedure stated previously, which comprised fitting an ANOVA model to the data, validating the method's assumptions, and employing an alternative non-parametric method if ANOVA was not appropriate.

#### 2.4.3. Comparison of the performance of distinct adaptive filtering methods and experimental conditions

##### 2.4.3.1. Evaluation based in time-domain features

The *GL*, *GH*, *GXin*, and *GXout* features were evaluated for both the EMG (*EMGr*) and EEG (*EEGr*) reference signals. *GL* and *GH* were used to evaluate the performance of adaptive filtering for each type of reference signal. In contrast to decomposition methods, the appropriate adaptive filtering method should reduce the signal amplitude in regions contaminated by EMG while preserving signal amplitude in regions of EEG without EMG contamination. Therefore, the appropriate adaptive filtering strategy for reducing the influence of EMG on EEG is the one that produces a *GL* close to zero (to preserve the EEG signal) and *GH* less than zero (indicating the reduction of EMG contamination).

The feature *GXin* and *GXout* were employed to evaluate and compare the behavior of the adaptive filtering in the regions of EEG with and without EMG. The lower the *GXout* compared to *GXin*, the greater the attenuation of the EEG regions contaminated by EMG. In addition, the closer to zero is *GXout* (*GXout* → 0), the greater the capacity of the adaptive filtering method to preserve the amplitude of the EEG signal in EMG-contaminated regions.

Box plots were used to visually investigate the values of central trend, dispersion and symmetry of the characteristics for each of the scenarios investigated. To verify the differences between the characteristics estimated from different adaptive filtering methods, the means of the variables were compared by ANOVA. If the premises of such a model were not verified, a non-parametric approach was then employed, as previously explained.

##### 2.4.3.2. Evaluation based in frequency-domain features

The non-filtered and filtered EEG signals were decomposed into their fundamental oscillations (Delta, Theta, Alpha, Beta, and Gamma) and box plots for the median frequency and its associated power were calculated. The study took into account the signal regions of the open and closed eye experimental conditions, as well as the overall signal that combines these two regions.

The normality of the variables were verified by the Kolmogorov–Smirnov test (*p* > 0.05), and then if the variables had a normal distribution the non-paired *t*-student test (*p* < 0.05) was applied to verify whether the mean of the variable related to the filtered signal reduced in relation to the non-filtered signal, i.e., the raw signal. If the distribution of the variables were not normal then the non-parametric Mann-Whitney U test was used (*p* < 0.05). Outliers were removed by eliminating observations that were outside of the following interval [*Q*1 − 1.5*IQR, Q*3 + 1.5*IQR*], in which *Q*1 is the first quartile, *Q*3 the third quartile and *IQR* the interquartile range.

#### 2.4.4. Comparison of execution time of decomposition and adaptive filtering methods

The execution time of the adaptive decomposition and filtering methods were evaluated by the means of the Matlab *timeit* function. This function performs multiple calls from the routine under analysis and returns the median value of the time measurements. The process for estimating the execution time employed actual and equal data for all methods. The evaluation considers samples with sizes from 25,000 to 1,000,000, with increments of 25,000, i.e., 40 different intervals. For each sample size, eight execution times were estimated to obtain a more representative estimate of the execution time.

The machine that processed the data had the following features: Ryzen 9 5900X 12-core/24-threads @3.7 GHz; RAM 2 × 16 GB DDR4 @ 3200 MHz; video card Asus RTX 3070 8 GB. The Matlab Parallel Computing Toolbox was used to run the applications in parallel, utilizing all of the computer's processors and available memory.

The comparison of execution time was performed by the non-parametric Kruskal–Wallis test (*p* < 0.05) as the Shapiro–Wilk test confirmed the distribution of the variables were not normal (*p* < 0.05). The pairwise comparison between variables was performed by the Nemenyi test (*p* < 0.05).

## 3. Results

### 3.1. Typical collected signals

**Figure 8:**
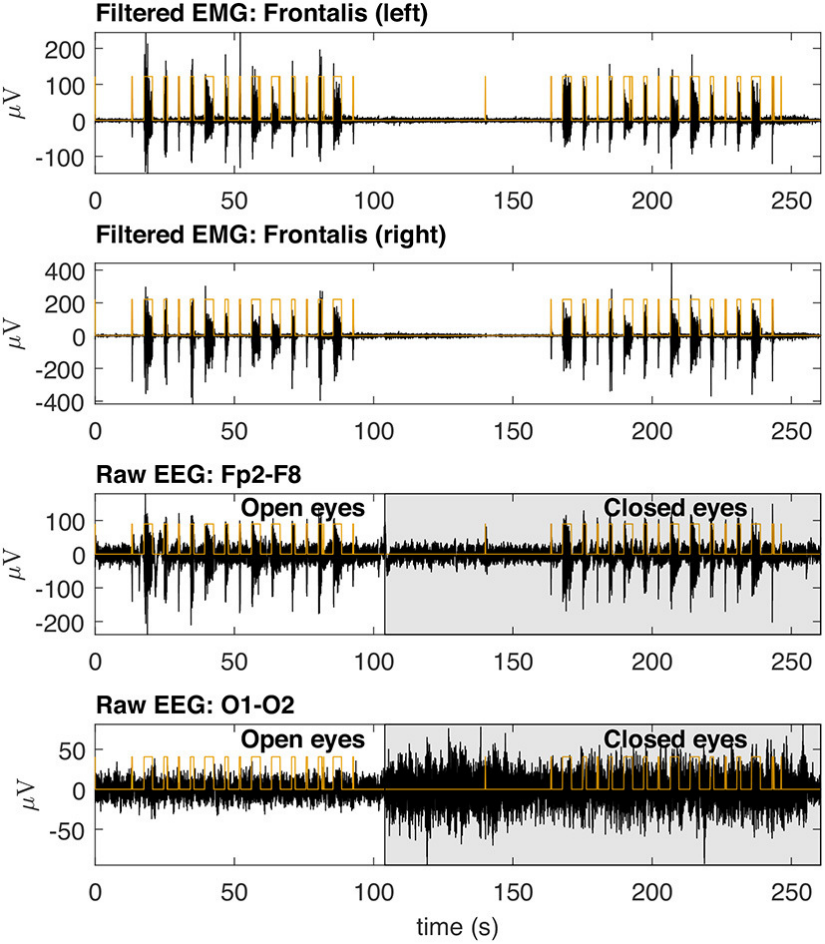
Typical EMG and EEG signals collected during the experimental trials. The EMG signals from the left and right Frontalis are shown. These signals were filtered to remove linear and non-linear trends. The EMG bursts were detected and then the binary signals oscillating from two levels were generated. Simultaneously collected EEG signals are shown for Fp2–F8 (high contamination) and O1-O2 (low contamination) locations. The binary signals are placed over the EEG signals for the indication of the periods in which there was EMG contamination.

Figure 8 shows typical EMG and EEG signals simultaneously collected during the experimental trials. The EMG bursts were detected by using the procedure described in Figure 4. The number of EMG bursts and their duration are in accordance to the protocol illustrated in Figure 2. The binary signals resulting from the EMG burst detection are plotted together with two typical EEG signals, one for the Fp2-F8 region, which is more contaminated, and the other for the O1-O2 region, which is less contaminated. Note that in the region when the eyes are closed (see the raw EEG: O1-O2) it is possible to see an increase in the amplitude of the EEG signal.

### 3.2. Characterization of the contamination of the EEG by EMG signals

**Figure 9:**
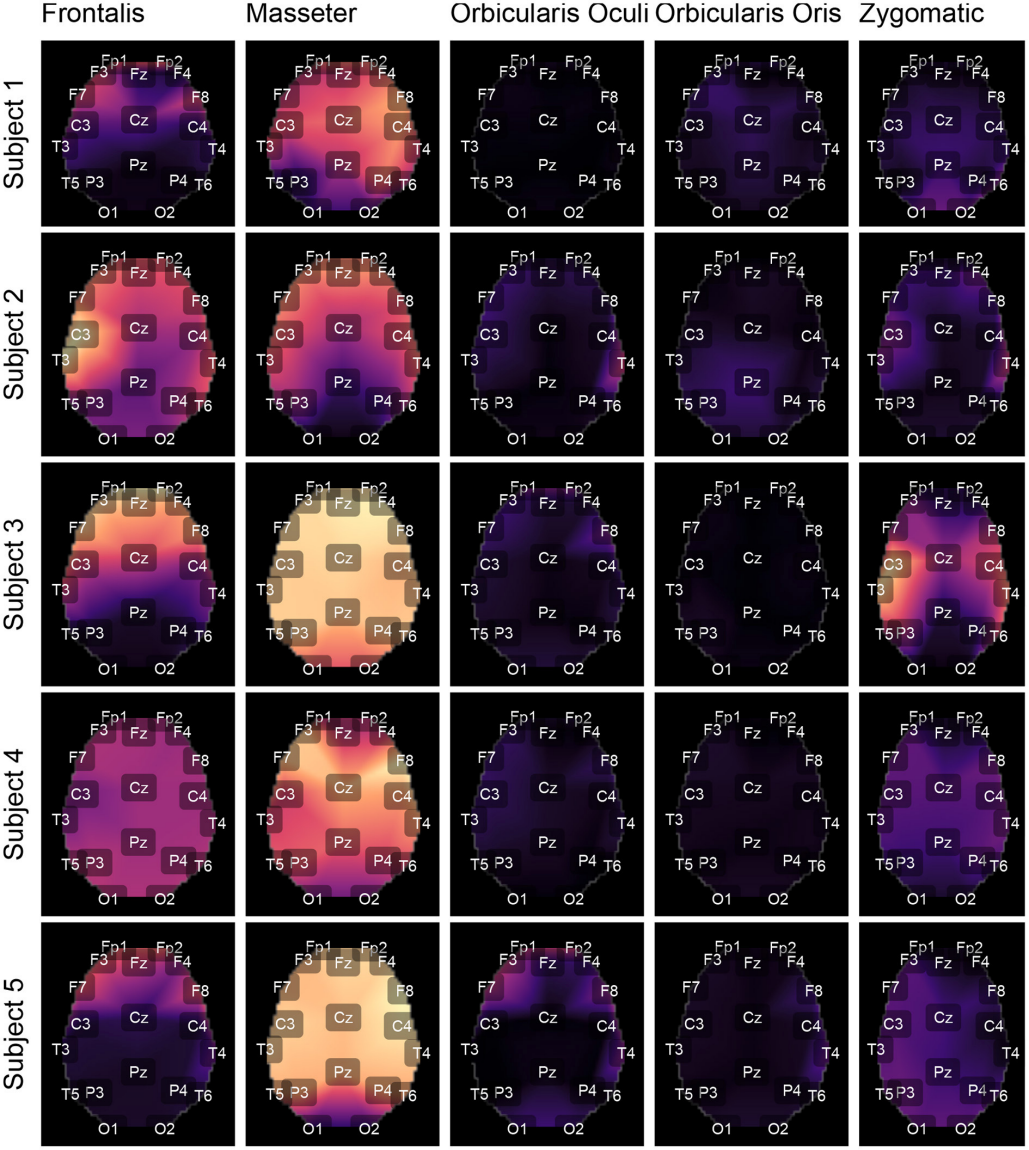
Using the international 10-20 system, the topological maps illustrate how the studied muscles contaminate distinct brain regions. Lighter colors represent the most contaminated locations, whereas darker colors denote the least contaminated areas. The presented results are for subjects from 1 to 5. The colors represent the similarity measure between the *GL* and *GH* features.

**Figure 10:**
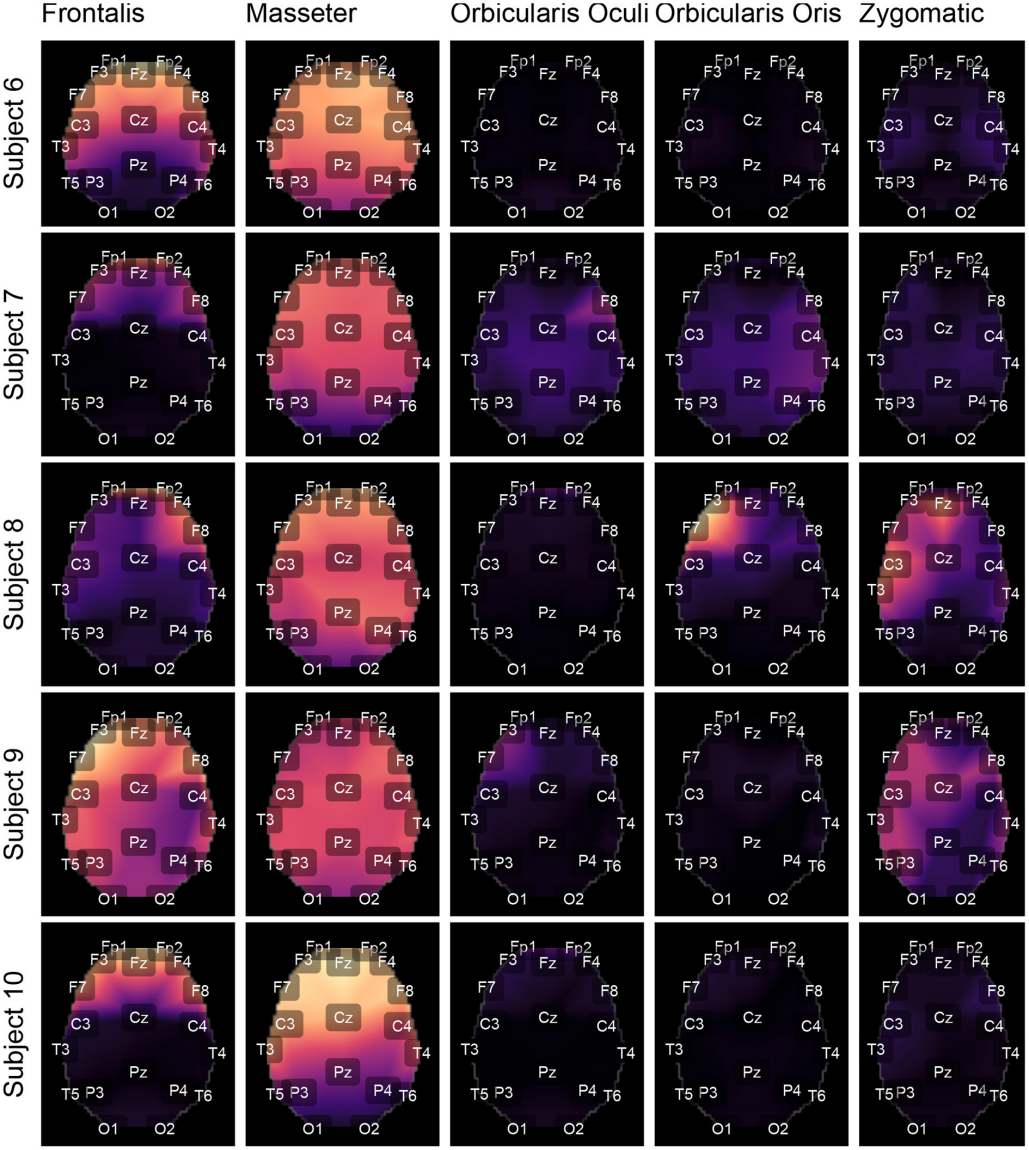
Topological maps for subjects from 6 to 10.

Figures 9, 10 depict topological maps for each subject and activated muscle. The visual inspection of the maps allow us to conclude that the Masseter is the muscle which produced the largest level of contamination, followed by the Frontalis and Zygomatic. These maps suggest also that the contamination and its spread over the brain map is dependent on the subject, which may be related to specific anatomical characteristics of the individual.

**Figure 11:**
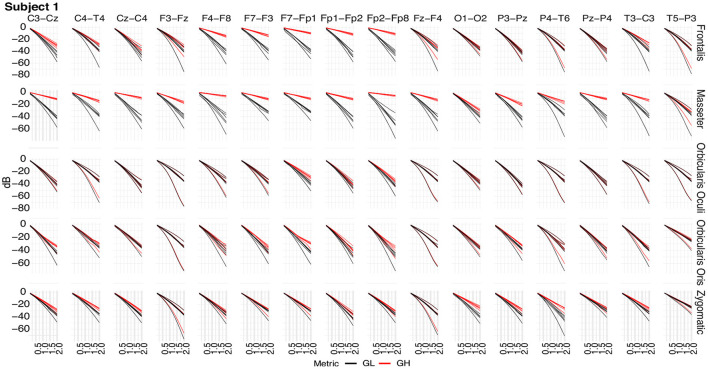
Typical *GL* and *GH* feature vectors estimated using different decomposition techniques for Subject 1. Each plot consists of six vector pairs, one pair for each method. The outcomes are presented for individual EEG sensors and muscles.

Figure 11 shows typical *GL* and *GH* feature vectors estimated for Subject 1. In each graphic six pairs of feature vectors are presented. Each pair of feature vectors was estimated from a specific decomposition method. The behavior of the feature vectors are similar for all subjects. The interpretation of the results is straightforward in the sense that the more similar the *GL* and *GH* feature vectors, the less contaminated the EEG signal is. For instance, for the occipital region (O1-O2) there is a high similarity (i.e., low distance) between the feature vectors for nearly all muscles, while for the frontal region (e.g., Fp1-Fp2) the produced contamination is higher for the Frontalis and Masseter. The estimates of feature vectors for the other subjects are available as a Supplementary Figures 2–10.

**Figure 12:**
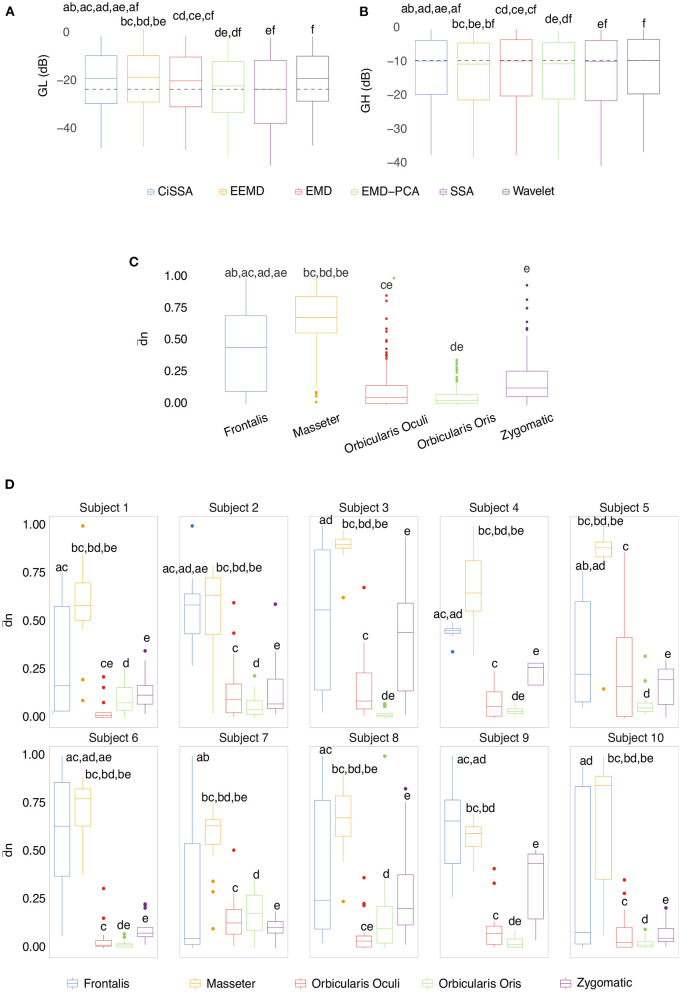
**(A)** Box plot of *GL* for distinct decomposition algorithms, regardless of subject and sensor location. The smaller the value of *GL*, the more appropriate the filtering method. The dashed lines represent the best result obtained with the SSA approach. Statistically significant differences between methods are represented by labels. All possible combinations of two were evaluated. For example, the EEMD method is represented by label “b” and was statistically different from CiSSA (label “a”), EMD (label “c”), EMD-PCA (label “d”), and SSA (label “e”). **(B)** Box plot of *GH* for distinct decomposition algorithms, regardless of subject and sensor location. The larger the value of *GH* the more suitable is the method for filtering. **(C)** Box plot of the mean normalized Euclidean distance between *GL* and *GH* for each muscle, independent of subjects and EEG sensors. The larger the value of this metric, the more contamination is caused by the muscle. **(D)** Box plot of the mean normalized Euclidean distance between *GL* and *GH* for each muscle and subject, independent of the EEG sensor.

Box plots of the mean of the normalized Euclidean distance between *GL* and *GH* vectors for each muscle are shown in Figures 12C,D. Figure 12C shows results that are independent of subjects and decomposition methods, whereas Figure 12D shows results that are dependent on subjects but independent of decomposition methods.

The results shown in Figures 12C,D are consistent with those observed in Figures 9, 10 (topological maps). In general, the Masseter was the muscle responsible for the highest EEG signal contamination, followed by the Frontalis and Zygomatic. The Frontalis exhibited the highest level of contamination variability, whereas the Orbicularis Oris produced the least. There was no significant difference between the mean normalized distances estimated from the Orbicularis Oculi and Orbicularis Oris muscles (Figure 12C). Figure 12D shows that a similar result was found for all subjects.

ANOVA could not be employed for the statistical analysis since its assumptions were violated. To compare the variables, the Kruskal–Wallis rank sum test and Dunn's test for multiple comparisons were used in all analyses.

### 3.3. Evaluation of decomposition methods to generate reference signals for adaptive filtering

In Figures 12A,B, the box plots of *GL* and *GH* are presented for each decomposition method, independently of subject and brain area. From the box plots, it is possible to compare and contrast the distributions of feature vectors for each investigated method. For *GL* the dashed lines indicate the median of the method which produces the largest amplitude reduction in the regions in which there is no EMG contamination. SSA was the most appropriate method among those considered.

For *GH*, the dashed lines represent the median of the approach that yields the lowest amplitude reduction in EMG-contaminated locations. Wavelet was the most appropriate method among those studied. In general, when both metrics, *GL* and *GH*, are considered, SSA is the most appropriate because it reduces the signal amplitude in regions without EMG contamination the most, while preserving the regions contaminated by EMG in a satisfactory manner, allowing the generation of an appropriate reference signal for adaptive filters. There was no statistically significant difference between the EEMD and Wavelet methods for *GL* (Figure 12A). There were no significant differences between CiSSA and EMD, and EEMD and EMD-PCA for *GH* (Figure 12B).

### 3.4. Evaluation of the filtering based on the time-domain features

**Figure 13:**
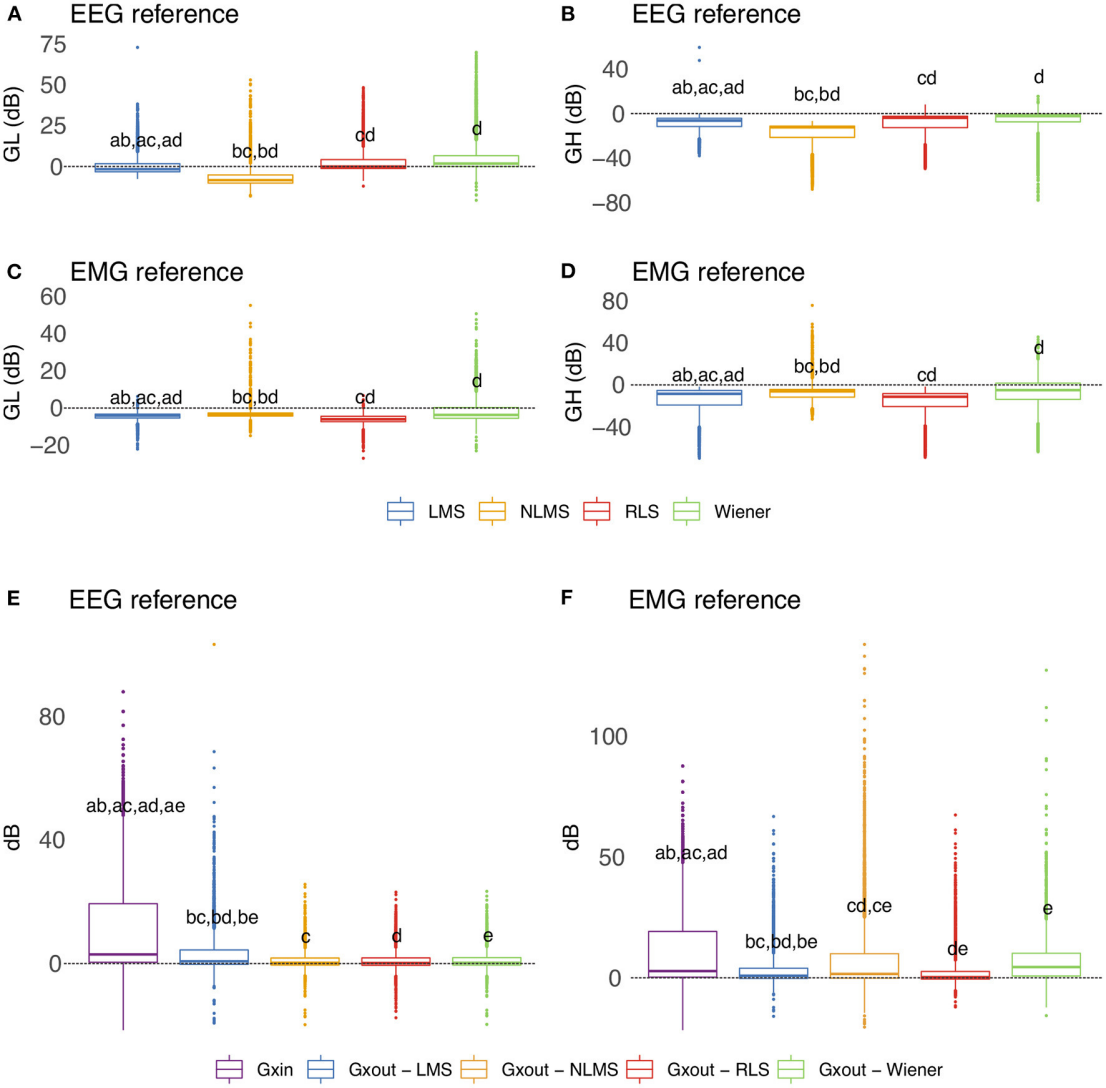
Evaluation of distinct adaptive filtering methods based on the time-domain features. The assessment is independent of the decomposition method and specific to the type of reference signal. **(A–D)** show results referent to *GH* and *GL*. **(E,F)** Present the results related to *GXin* and *GXout*.

Figure 13 depicts the overall behavior of distinct adaptive filtering methods according to the time-domain features and type of reference signals (EEG or EMG). In general, all adaptive filtering methods were capable of filtering EMG contamination from EEG. As can be seen in Figures 13B,D, the medians of *GH* are less than zero, confirming, thus, that the EMG contamination was attenuated. On the other hand, when evaluating the preservation of regions of EEG without EMG, the results vary, depending on the type of reference. Ideally the median of *GL* should be as close to zero as possible. When the reference signal is the EEG, the most appropriate method is the RLS as it causes the lowest changes in the regions of EEG without EMG contamination. When the reference is the EMG signal, the most appropriate methods were the NLMS and Wiener.

Figures 13E,F show the behavior of the variables *GXin* and *GXout* for adaptive filtering. For the EEG reference, the obtained results confirm the attenuation of the EMG signal, as all the medians of *Xout* are lower than the median of *GXin*. On the other hand, for the EMG reference, the results yielded by the Wiener filter were not satisfactory.

**Figure 14:**
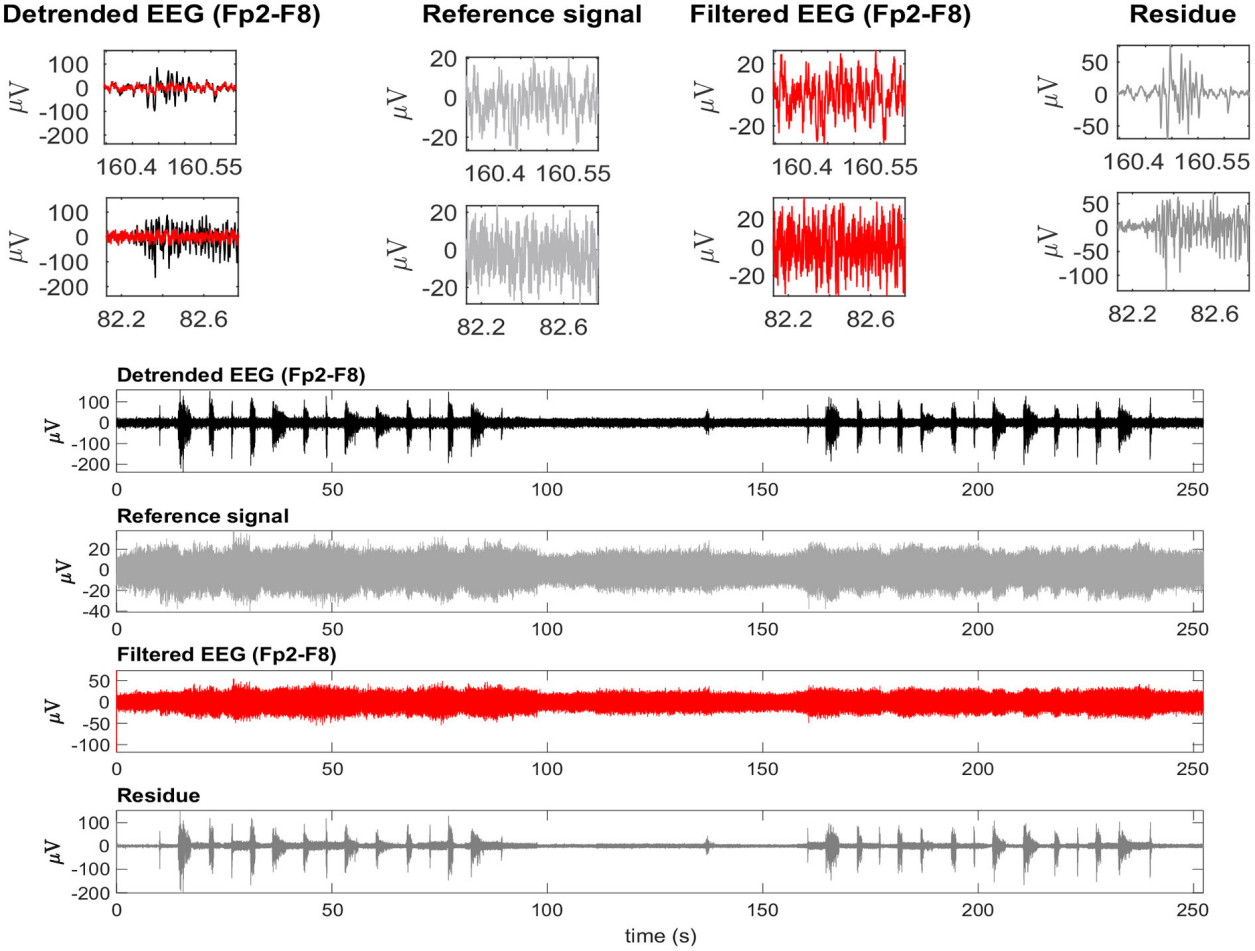
A typical EEG signal corrupted by facial EMG. The EEG signal is from the Fp2-F8 region because it is most affected by facial electromyography. EMG bursts can be seen on the detrended EEG signal. The EEG signal was used as a reference signal, estimated from EMD, and then filtered using the RLS filter. The residue, which is the difference between the detrended and filtered EEG data, clearly shows the EMG activity that was eliminated from the signal. The inset plots at the top indicate the selection of two EEG regions contaminated by EMG. The filtered signal is shown over the contaminated signal in red. For each region, the detrended EEG, reference signal, filtered EEG, and residue are shown.

Considering the time-domain features, the EEG reference signal was more appropriate for the adaptive filtering, as it allowed for the preservation of EEG regions not contaminated by EMG. Furthermore, when evaluating the variables *GXin* and *GXout* it is clear that when using the EEG as a reference, the filtered EMG-contaminated region will preserve the EEG activity. Figure 14 shows an example of EMG-corrupted EEG and its filtered version. The figure insets show the effect of the adaptive filtering, in which the EMG amplitude is attenuated and the EEG activity in the EMG-contaminated region follows the EEG dynamics of the EEG signal in the neighbor regions in which there are no EMG contamination.

### 3.5. Evaluation of the filtering based on the frequency domain features

**Figure 15:**
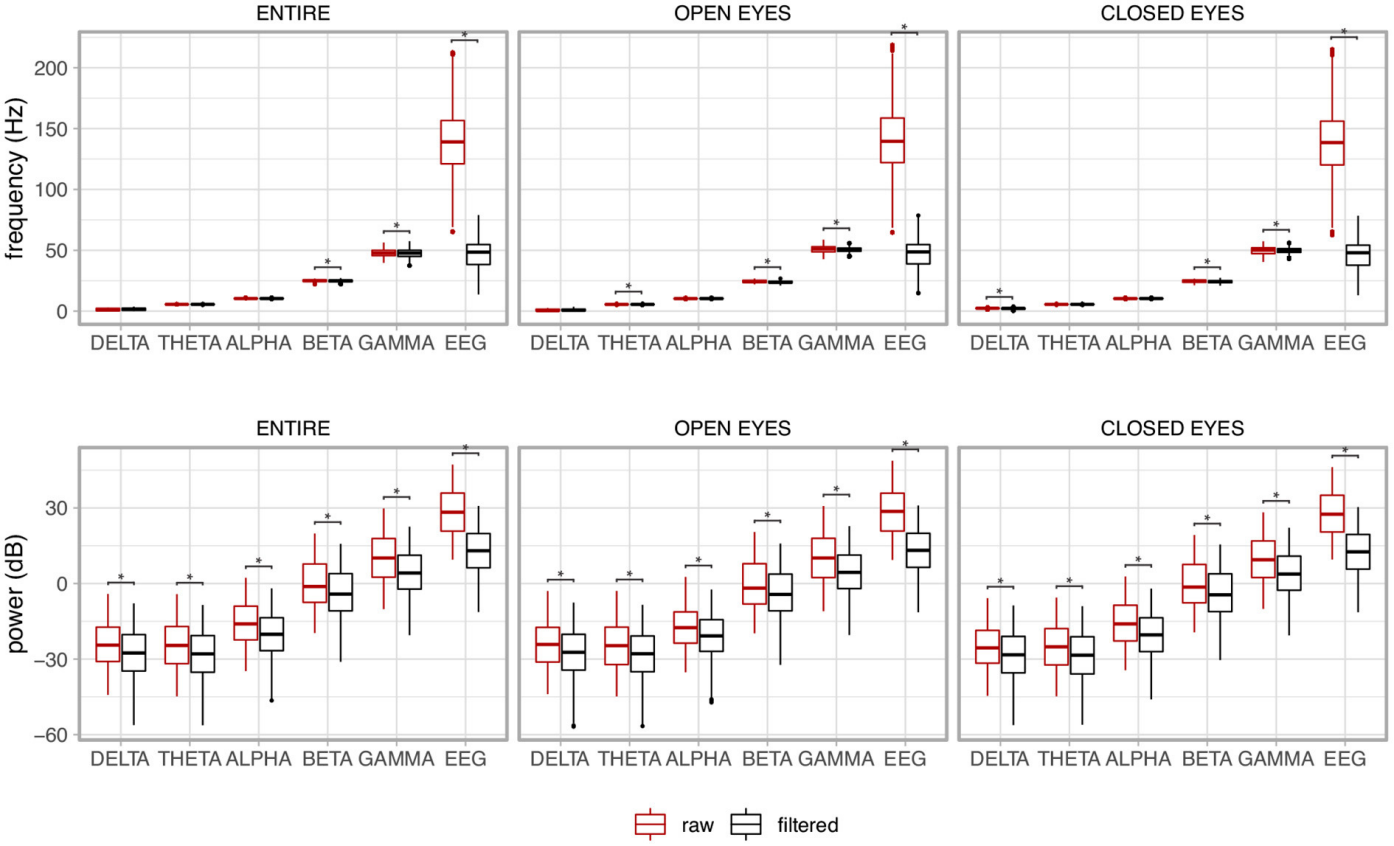
Median frequency and its power of the electroencephalogram (EEG) together with its components for the whole signal (ENTIRE) and the two experimental conditions (OPEN EYES and CLOSED EYES). A contrast between the raw non-filtered signal with the filtered signal is presented. The asterisks show the pair of variables in which the variable associated to the filtered signal was significantly reduced in comparison to the non-filtered signal, i.e., raw signal.

The box plots in Figure 15 show the behavior of the median frequency and its power for the EEG signal together with its components, i.e., Delta (0.5–4 Hz), Theta (4–7 Hz), Alpha (7–13), Beta (13–30), and Gamma (30–70 Hz). The results contrast the raw non-filtered signal with the filtered signal. The non-parametric Mann-Whitney U test was used as the distributions of the variables were not normal (*p* < 0.05).

There is a clear drop in the frequency and power of the EEG signal resulting from the filtering. As expected, the power of the signal is reduced for all components although their median frequencies are kept in the expected frequency band. The same behavior is noted for the experimental conditions of open and closed eyes, and for the whole signal, which considers the joint analysis of the open and closed eye regions.

### 3.6. Execution time of decomposition and adaptive filtering methods

**Figure 16:**
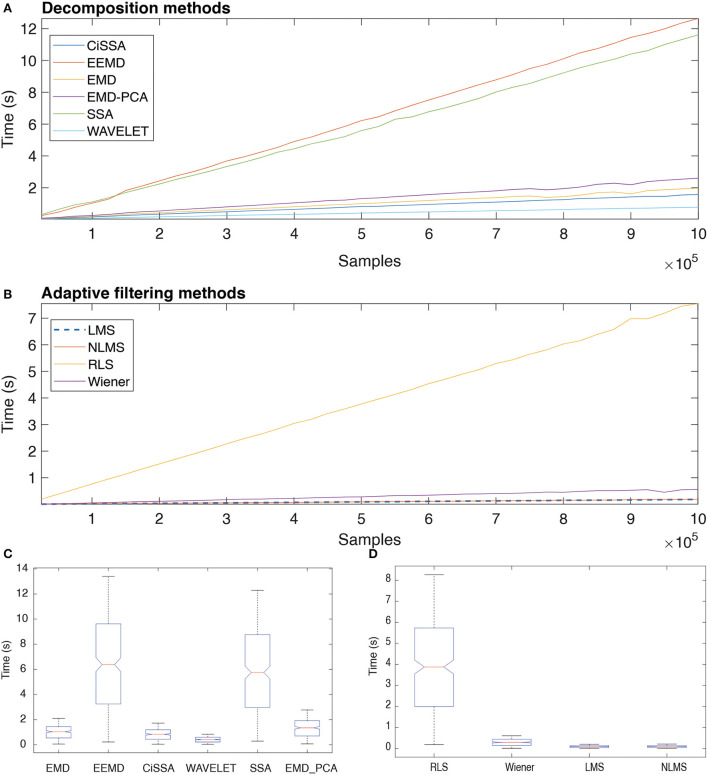
Execution time of distinct decomposition **(A)** and adaptive filtering **(B)** methods as function of the number of samples. The box plots show the central trend and dispersion of execution times **(C,D)**.

Figure 16 depicts the typical execution times for all methods investigated. The estimates in A and B are based on the mean of the eight execution times for each sample size. The linear relationship between sample size and execution time may indicate that the methods have linear computational complexity, *O*(*n*).

According to the results in Figure 16C, the execution times for EEMD and SSA are statistically equivalent (Nemenyi Test, *p* > 0.05), just as they are for EMD and CiSSA. Wavelet obtained the shortest execution time. According to the results in Figure 16D, the execution times for LMS and NLMS are statistically equivalent. Furthermore, RLS has a significantly longer execution time than the others.

## 4. Discussion

The filtering approach depicted in Figure 3 has been proposed for the removal of facial muscular artifacts from EEG signals, which is an important requirement for distinct applications. It is a single channel approach for filtering low SNR EEG signals. In general, the proposed method is based on a hybrid filtering approach, combining adaptive filters with decomposition techniques. Thus, in this research, the performance of several decomposition (EMD, EEMD, CiSSA, Wavelet, SSA, and EMD-PCA) and optimal filtering (RLS, Wiener, LMS, and NMLS) methods were evaluated.

Although it is possible to find some databases containing EEG signals corrupted by EMG, we could not find any open data set similar to the one that was collected in this research. The relevance of the collected data set is that it considers the influence of the activity of several facial muscles to the contamination of EEG signals. The experimental protocol was carefully designed to take into consideration data collection in practical scenarios, such as the execution of facial expressions commonly used for some human-computer interfaces based on facial EMG (Andrade et al., 2013). In addition, all EEG signals were collected simultaneously with EMG signals guaranteeing the necessary synchronization between signals and the possibility of annotating the regions in which EEG signals were actually contaminated by EMG. The dataset included a total of 2,250 s of EEG signals corrupted by EMG, with the participation of 10 subjects and distinct experimental conditions (e.g., open and closed eyes, EMG bursts of varying durations, and the activation of different facial muscles), allowing for the required variability to test the performance of filtering methods.

Other decomposition and adaptive filtering methods can be added to the filter architecture of the EEG single channel filtering approach depicted in Figure 3 without altering the entire filtering strategy. This is an interesting feature for the development of computational libraries that can benefit from the use of encapsulated code implementing decomposition and adaptive filtering methods that can be directly plugged into the general steps, i.e., the processing pipe depicted in Figure 3. To aid in the diffusion of this architecture, the authors of this paper have made available all Matlab and R scripts at https://doi.org/10.5281/zenodo.6591866. In addition, sample data and demonstration scripts are provided to facilitate comprehension and replication of the filtering approach presented and evaluated in this study.

In this study, the detection of EMG bursts is relevant because these bursts mark the regions contaminated and non-contaminated by electromyography automatically. The identification of these regions are used in the stage of soft-thresholding signal components and for computing the proposed set of features to measure the performance of filtering methods. We decided not to detect the bursts directly from the EEG contaminated signal to guarantee that the noise present in the EEG signal was really from the EMG activity. However, the use of the proposed filtering approach (Figure 3) can be applied without the simultaneously collection of EMG signals. If this is the case, it would be necessary to detect EMG bursts directly from the EMG-corrupted EEG.

The time and frequency domain features proposed in this work (Figure 5) were advantageous for the characterization of facial EMG contamination on the EEG. Figures 9, 10 depict an approach for energy visualization of topological maps displaying the degree of contamination created by distinct muscles based on this set of characteristics. Utilizing the normalized Euclidean distance as a measure of similarity allowed for the visual, qualitative, and quantitative comparison of topological maps estimated for various subjects and active muscles.

As indicated in Figure 3 an important step for adaptive filtering is the generation of reliable reference signals. In the proposed approach the reference signal is generated from the application of soft-thresholding to the signal components. To evaluate the performance of distinct decomposition methods the *GL* and *GH* metrics were proposed. These metrics were also employed to characterize the EMG contamination in distinct regions of the scalp (Figures 9, 10). An interesting aspect of the contamination is that although there is a general pattern, e.g., muscles such as the Frontalis and Masseter contributed more to the EEG contamination, the way this contamination spread over the scalp is specific to the individual. This fact can be verified by the relatively large variability of the variables presented in Figures 12C,D.

According to the findings (Figures 9, 10, 12), the Masseter muscle provided the highest degree of contamination, followed by the Frontalis and Zygomatic. Variability between individuals was an important component of this study; for example, the Orbicularis Oris of subject 8 produced a substantial contamination of the EEG obtained in the frontal region. This may involve anatomical and behavioral characteristics of the individual. This requires that EEG filtering methods be devised to accommodate data variations introduced by anatomical, physiological, and experimental settings. This also justifies the more sophisticated experimental methodology utilized in this study.

In general, all decomposition methods investigated in this study were suitable for generating adequate adaptive filtering reference signals. Nonetheless, we believe that the SSA method is superior because it successfully preserved EEG in non-contaminated regions while lowering the signal amplitude in EMG-contaminated regions significantly (Figures 12A,B). Considering the type of signal reference (EMG or EEG) the results reported in Figure 13 suggest that the reference based on the EEG signal is preferable because of the lower variability exhibited (Figures 13E,F) when compared to the EMG reference.

This study also investigated various adaptive filtering algorithms (LMS, NLMS, RLS, and Wiener) to reduce electromyographic activity as much as possible in EEG data heavily contaminated by EMG. When using the EEG as a reference signal, RLS and NLMS were the best methods among those tested. In general, these methods best ensured: (i) attenuation of electromyographic activity in regions of EMG-contaminated EEG (Figure 13B), (ii) preservation of electroencephalographic activity in regions of EEG without EMG (Figure 13A), and (iii) the dynamics of the resulting signal, not mischaracterizing the EEG signal over time (Figures 13, 14). However, when the reference signal was the EMG, the LMS, and RLS algorithms performed best. Thus, the RLS method is the most preferred, as it produced satisfactory results regardless of the type of reference signal.

The results shown in Figure 15 confirm that the single channel approach proposed in this research was capable of reducing EMG contamination on the EEG signals, while preserving relevant information of the electroencephalogram. For instance, typical values of frequency were found for each EEG component. In addition, there was reduction in the power of the non-filtered EEG signal compared to its filtered version, and this could also be observed for each EEG component.

The results presented in Figure 16 reveal that there is a linear relationship between the number of samples, i.e., signal length, and the execution time required to process the data. This suggests that the decomposition and filtering algorithms have a linear computational complexity. In total, 90 h were spent processing the entire data set of this investigation, taking into account the processing time for ten subjects and all experimental conditions. Methods that need less time to process data are desirable in this regard, even if they do not produce optimal results.

In future research, the proposed method could be utilized to reduce interference produced by electromyography in applications controlled by electroencephalographic activity (such as brain-computer interfaces). Furthermore, while this study only involves healthy people, we encourage the examination and confirmation of the technique created for people with disorders like amyotrophic lateral sclerosis. This would allow for the creation of more robust assistive technology as well as a better understanding of the electroencephalographic activity associated with this type of clinical condition.

## 5. Conclusion

This study introduced a single-channel filtering method for reducing facial electromyography from EEG signals. The proposed method is sufficiently general to accommodate multiple decomposition and adaptive filtering techniques within a single architecture.

Using a data set that enabled the generation of EMG-corrupted EEG in experiments involving facial muscular activation, the filtering method was evaluated. The set of time and frequency domain characteristics enabled the visualization and quantification of facial EMG contamination of the EEG. This set of features allowed for comparative analysis of filtering methods.

The results indicated that the Masseter was the muscle that contaminated the EEG the most; however, individual variation should not be disregarded, as the contraction of other facial muscles in some people may generate significant contamination on EEG signals.

In general, all investigated decomposition and adaptive filtering methods effectively filtered facial EMG-corrupted EEG; however, the decomposition method SSA reduced EMG contamination while preserving the EEG signal more. This method's relative slowness in comparison to other studies is its most significant drawback. In terms of the adaptive filtering method, it was observed that the reference signal (EMG or EEG) affects the method's performance, despite the methods' similarities.

## Data availability statement

The datasets presented in this article are not readily available because, the set of features can be shared, but no the raw data, as this is not allowed by the Brazilian regulations. Requests to access the datasets should be directed to https://doi.org/10.5281/zenodo.6591866.

## Ethics statement

This study follows the Resolution 466/2012 of the National Health Council. The study was conducted at the Centre for Innovation and Technology Assessment in Health of the Federal University of Uberlândia (UFU), Brazil. The protocols were approved by the Human Research Ethics Committee (CEP-UFU), CAAE Number: 43670815.4.0000.5152. The patients/participants provided their written informed consent to participate in this study. Written informed consent was obtained from the individual(s) for the publication of any potentially identifiable images or data included in this article.

## Author contributions

All authors listed have made a substantial, direct, and intellectual contribution to the work and approved it for publication.

## Funding

The present work was carried out with the support of the National Council for Scientific and Technological Development (CNPq), Coordination for the Improvement of Higher Education Personnel (CAPES—Program CAPES/ DFATD-88887.159028/ 2017-00, Program CAPES/ COFECUB-88881.370894/ 2019-01, and Program CAPES-PRINT-UFU) and the Foundation for Research Support of the State of Minas Gerais. AO is fellow of CNPq, Brazil (304818/2018-6 and 305223/2014-3). MV is fellow of CNPq, Brazil (304533/2020-3). AP is fellow of CNPq, Brazil (309525/2021-7).

## Conflict of interest

The authors declare that the research was conducted in the absence of any commercial or financial relationships that could be construed as a potential conflict of interest.

## Publisher's note

All claims expressed in this article are solely those of the authors and do not necessarily represent those of their affiliated organizations, or those of the publisher, the editors and the reviewers. Any product that may be evaluated in this article, or claim that may be made by its manufacturer, is not guaranteed or endorsed by the publisher.
