# Supplementary material for: Single channel approach for filtering electroencephalographic signals strongly contaminated with facial electromyography
Source: Front Comput Neurosci. 2022 Jul 26;16:822987. doi: 10.3389/fncom.2022.822987 (PMC9361713; doi:10.3389/fncom.2022.822987)
Supplement: Supplementary file 1 [file Data_Sheet_1.PDF]

Supplementary Material

1 SUPPLEMENTARY FIGURES

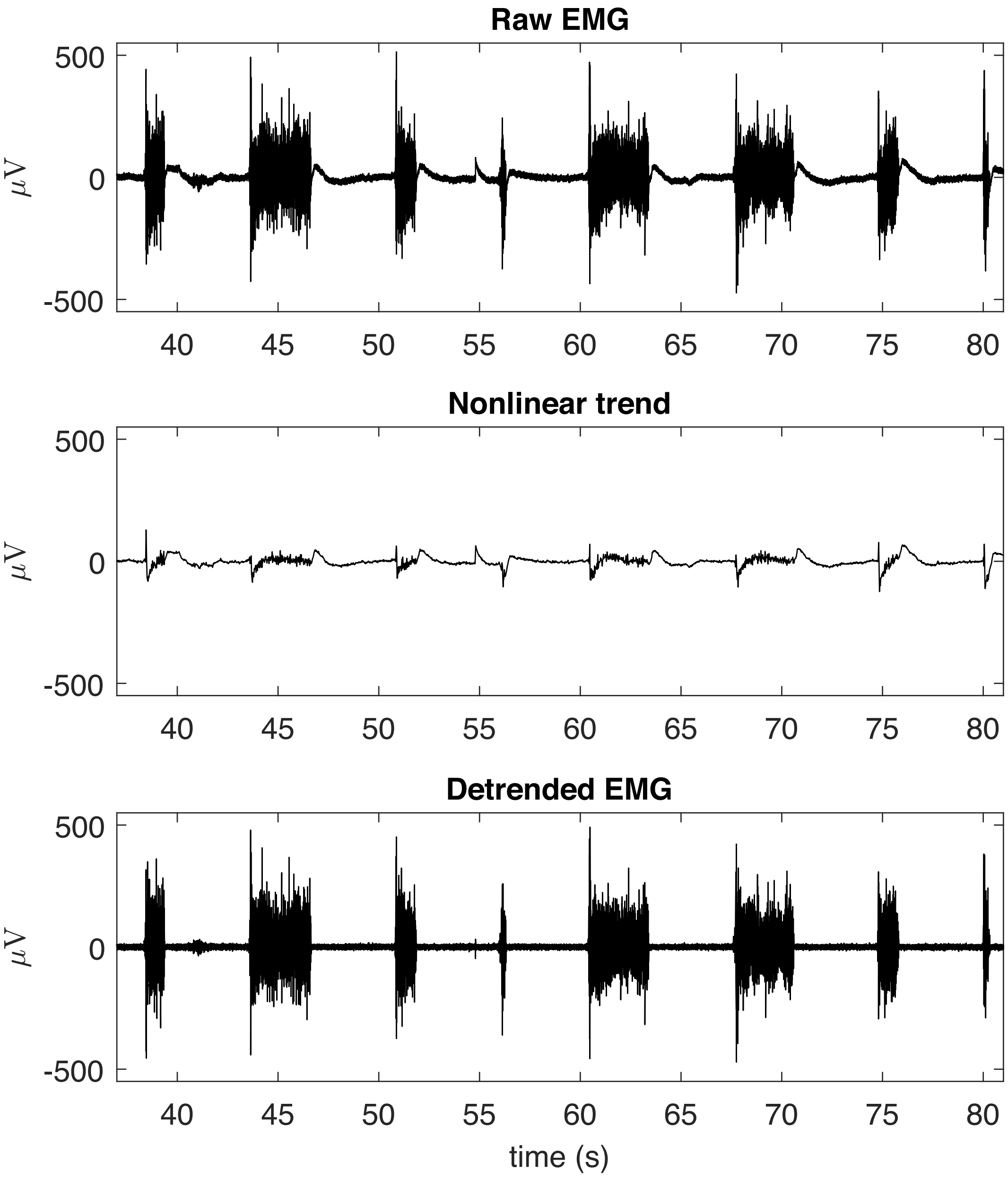

Figure S1. The raw EMG signal is corrupted by linear and nonlinear trends. The estimated nonlinear trend and the detrended signals are shown.

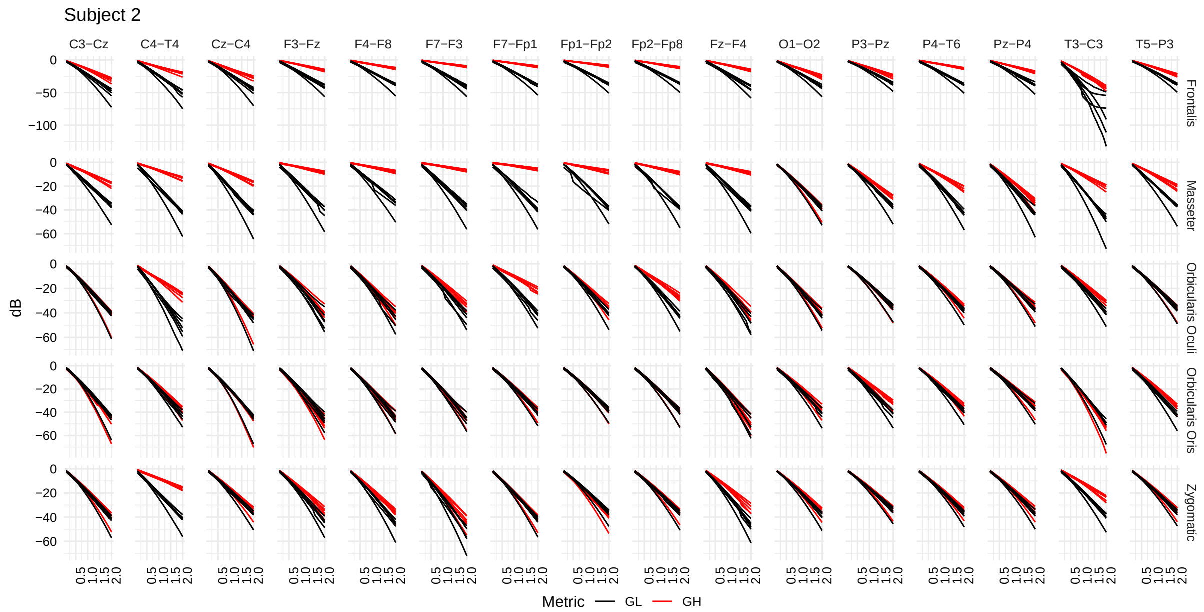

Figure S2. Typical GL and GH feature vectors estimated using different decomposition techniques for Subject 2. Each plot consists of six vector pairs, one pair for each method. The outcomes are presented for individual EEG sensors and muscles.

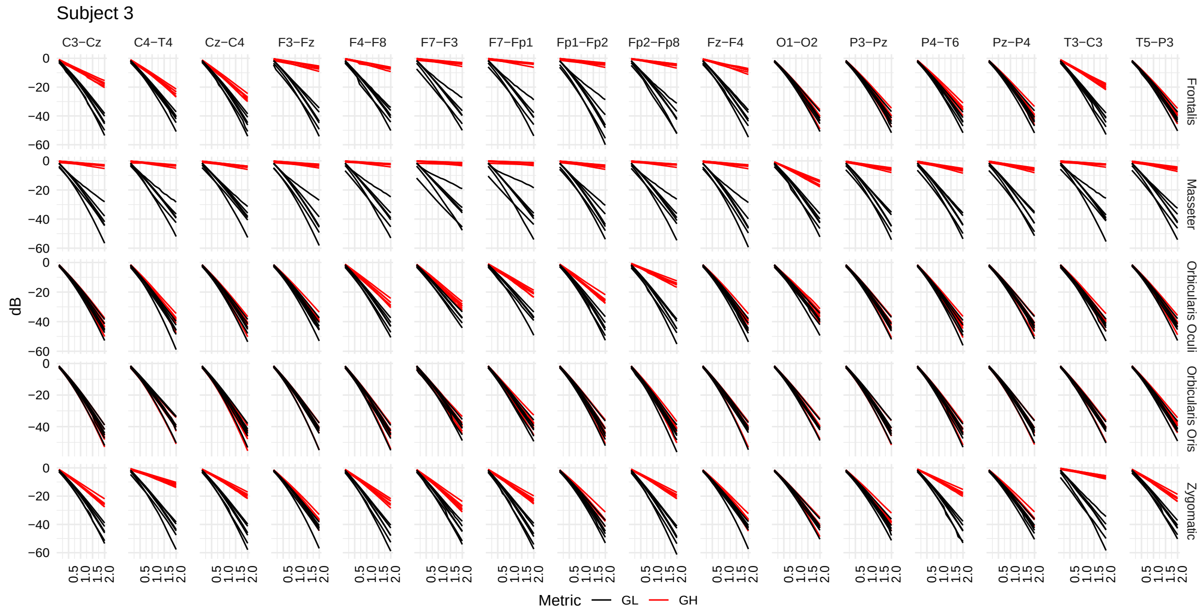

Figure S3. Typical GL and GH feature vectors estimated using different decomposition techniques for Subject 3. Each plot consists of six vector pairs, one pair for each method. The outcomes are presented for individual EEG sensors and muscles.

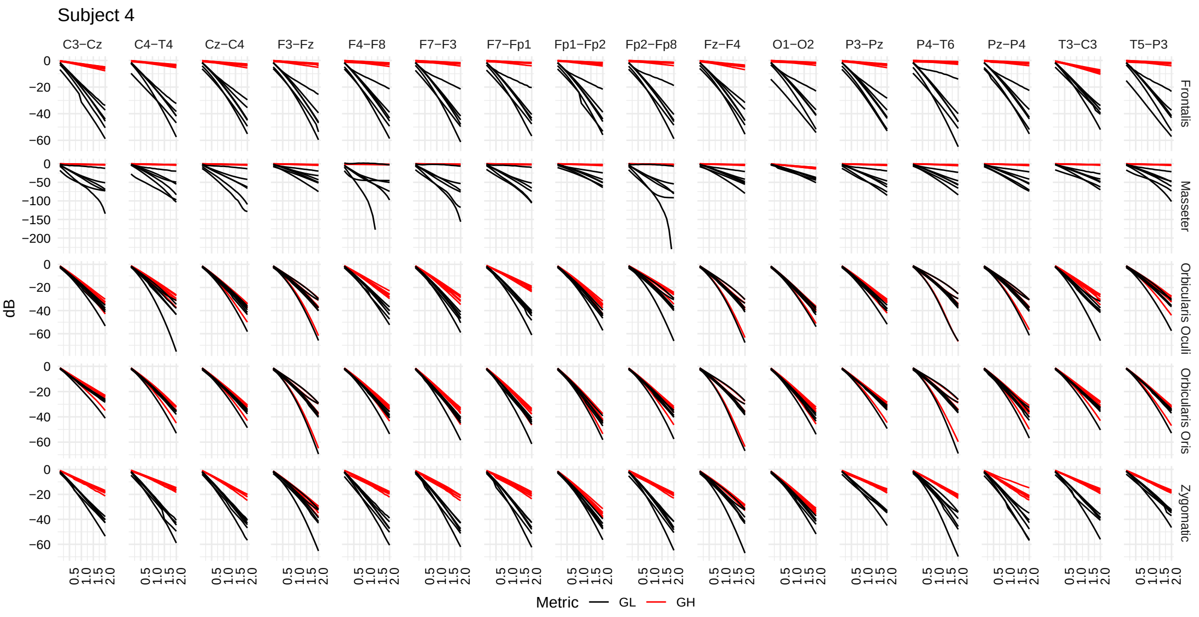

Figure S4. Typical GL and GH feature vectors estimated using different decomposition techniques for Subject 4. Each plot consists of six vector pairs, one pair for each method. The outcomes are presented for individual EEG sensors and muscles.

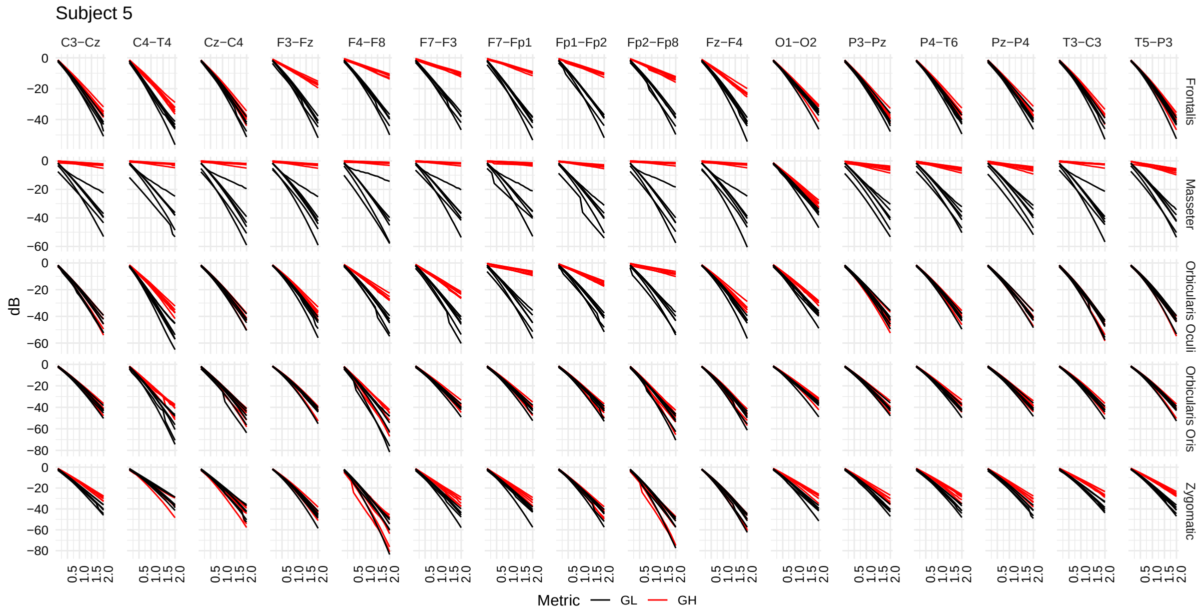

Figure S5. Typical GL and GH feature vectors estimated using different decomposition techniques for Subject 5. Each plot consists of six vector pairs, one pair for each method. The outcomes are presented for individual EEG sensors and muscles.

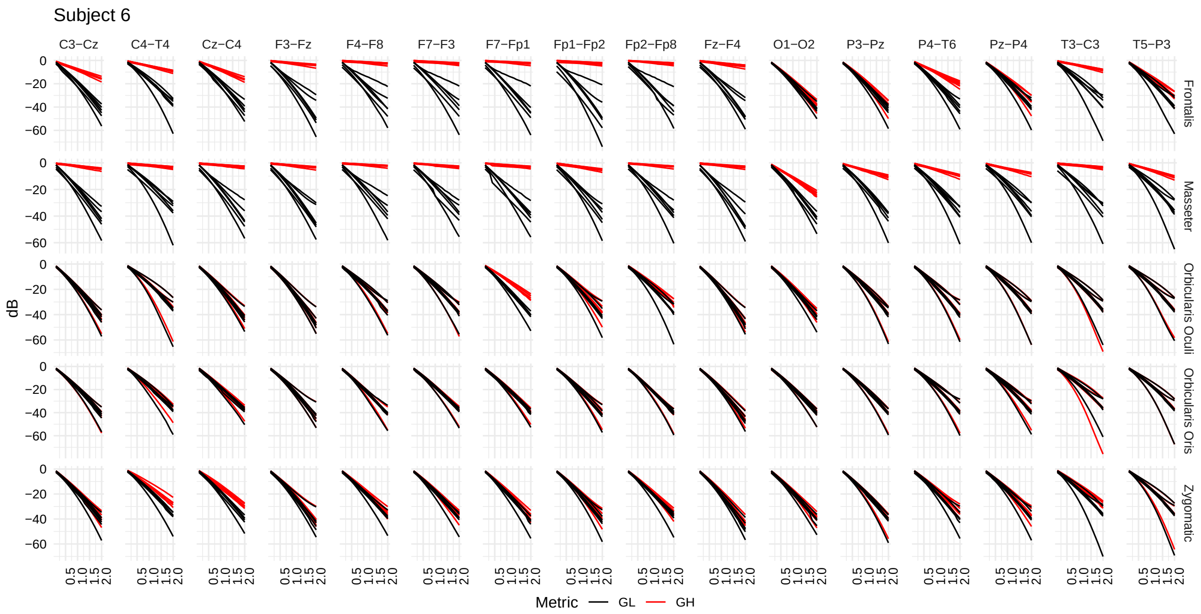

Figure S6. Typical GL and GH feature vectors estimated using different decomposition techniques for Subject 6. Each plot consists of six vector pairs, one pair for each method. The outcomes are presented for individual EEG sensors and muscles.

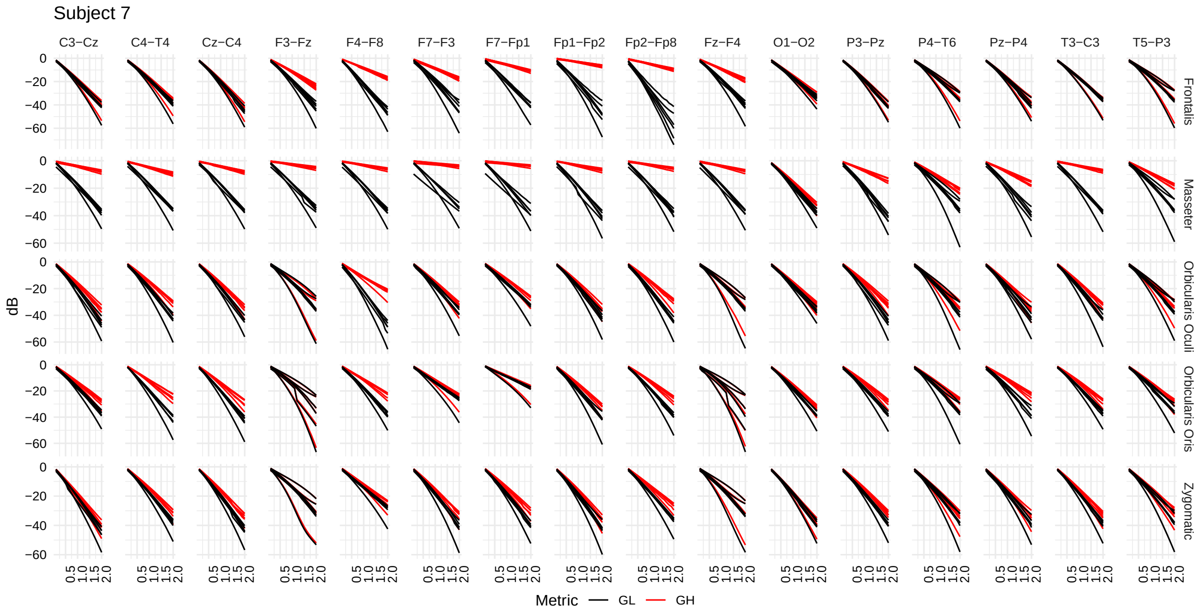

Figure S7. Typical GL and GH feature vectors estimated using different decomposition techniques for Subject 7. Each plot consists of six vector pairs, one pair for each method. The outcomes are presented for individual EEG sensors and muscles.

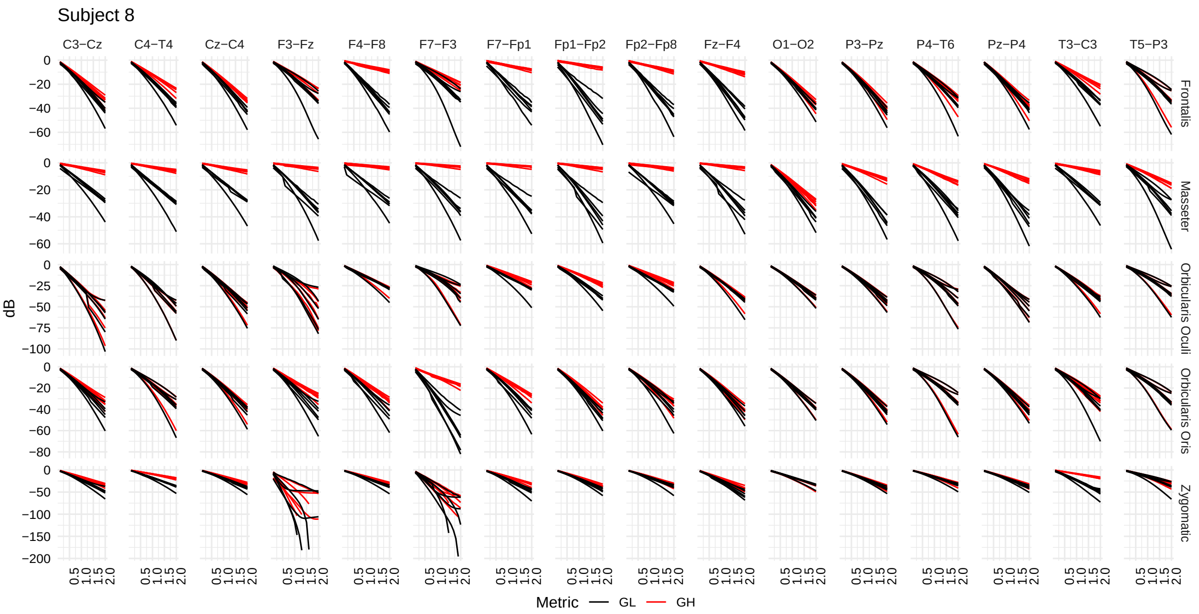

Figure S8. Typical GL and GH feature vectors estimated using different decomposition techniques for Subject 8. Each plot consists of six vector pairs, one pair for each method. The outcomes are presented for individual EEG sensors and muscles.

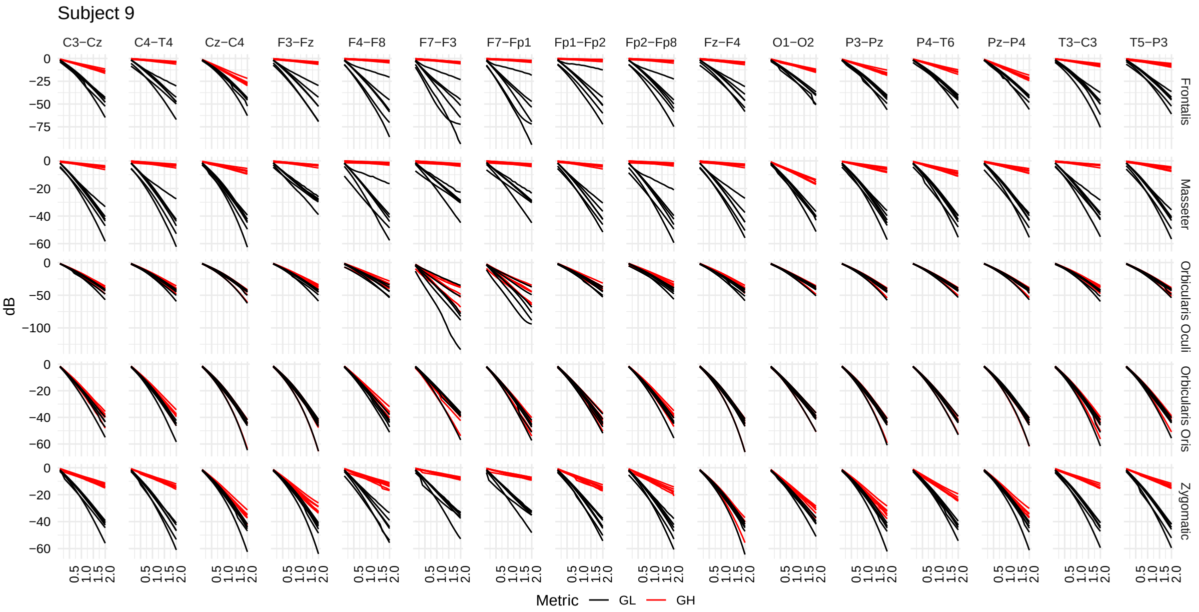

Figure S9. Typical GL and GH feature vectors estimated using different decomposition techniques for Subject 9. Each plot consists of six vector pairs, one pair for each method. The outcomes are presented for individual EEG sensors and muscles.

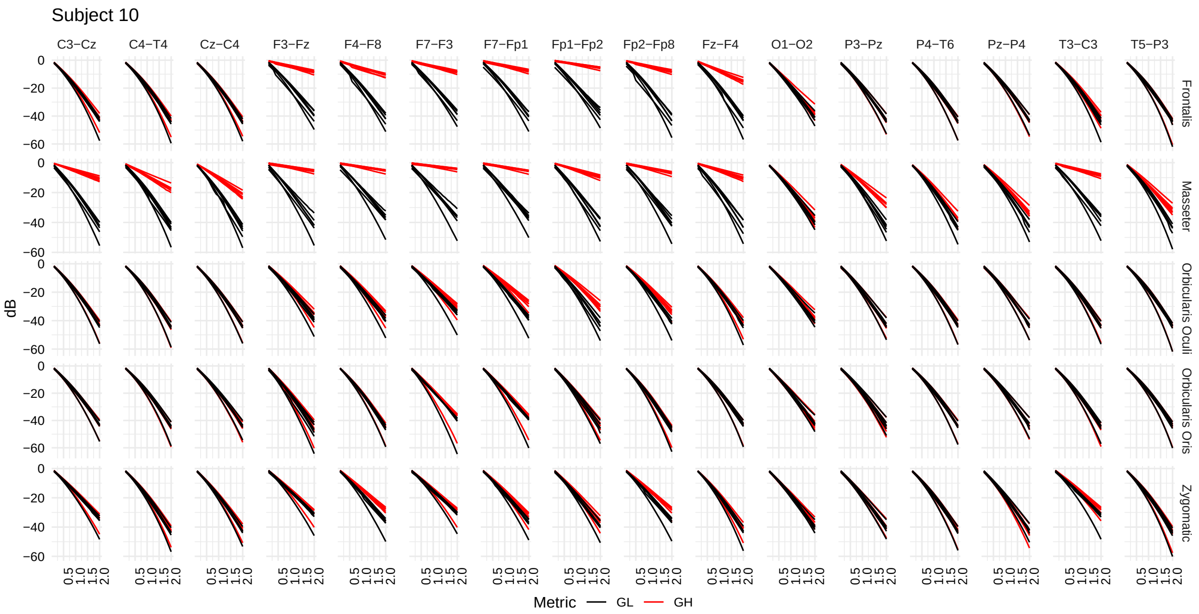

Figure S10. Typical GL and GH feature vectors estimated using different decomposition techniques for Subject 10. Each plot consists of six vector pairs, one pair for each method. The outcomes are presented for individual EEG sensors and muscles.
